# Ion Permeabilities in Mouse Sperm Reveal an External Trigger for SLO3-Dependent Hyperpolarization

**DOI:** 10.1371/journal.pone.0060578

**Published:** 2013-04-05

**Authors:** Julio C. Chávez, José L. de la Vega-Beltrán, Jessica Escoffier, Pablo E. Visconti, Claudia L. Treviño, Alberto Darszon, Lawrence Salkoff, Celia M. Santi

**Affiliations:** 1 Departamento de Genética del Desarrollo y Fisiología Molecular, Instituto de Biotecnología, Universidad Nacional Autónoma de México, Cuernavaca, Morelos, México; 2 Department of Anatomy and Neurobiology, Washington University School of Medicine, St. Louis, Missouri, United States of America; 3 Department of Genetics, Washington University School of Medicine, St. Louis, Missouri, United States of America; 4 Department of Veterinary and Animal Science, Integrated Sciences Building, University of Massachusetts, Amherst, Massachusetts, United States of America; Cornell University College of Veterinary Medicine, United States of America

## Abstract

Unlike most cells of the body which function in an ionic environment controlled within narrow limits, spermatozoa must function in a less controlled external environment. In order to better understand how sperm control their membrane potential in different ionic conditions, we measured mouse sperm membrane potentials under a variety of conditions and at different external K^+^ concentrations, both before and after capacitation. Experiments were undertaken using both wild-type, and mutant mouse sperm from the knock-out strain of the sperm-specific, pH-sensitive, SLO3 K^+^ channel. Membrane voltage data were fit to the Goldman-Hodgkin-Katz equation. Our study revealed a significant membrane permeability to both K^+^ and Cl^−^ before capacitation, as well as Na^+^. The permeability to both K^+^ and Cl^−^ has the effect of preventing large changes in membrane potential when the extracellular concentration of either ion is changed. Such a mechanism may protect against undesired shifts in membrane potential in changing ionic environments. We found that a significant portion of resting membrane potassium permeability in wild-type sperm was contributed by SLO3 K^+^ channels. We also found that further activation of SLO3 channels was the essential mechanism producing membrane hyperpolarization under two separate conditions, 1) elevation of external pH prior to capacitation and 2) capacitating conditions. Both conditions produced a significant membrane hyperpolarization in wild-type which was absent in SLO3 mutant sperm. Hyperpolarization in both conditions may result from activation of SLO3 channels by raising intracellular pH; however, demonstrating that SLO3-dependent hyperpolarization is achieved by an alkaline environment alone shows that SLO3 channel activation might occur independently of other events associated with capacitation. For example sperm may undergo stages of membrane hyperpolarization when reaching alkaline regions of the female genital tract. Significantly, other events associated with sperm capacitation, occur in SLO3 mutant sperm and thus proceed independently of hyperpolarization.

## Introduction

Fertilization involves the fusion of male and female gametes which is the first step in creating a new individual. Mammalian sperm encounter environments with very different ionic composition on their journey to meet the egg. For example external K^+^ concentration ([K^+^]_e_) may change from ∼39 to 5–8 mM, external Cl^−^ concentration ([Cl^−^]_e_) from ∼27 to 130 mM, and external Na^+^ concentration ([Na^+^]_e_) from 38 to 140 mM in the cauda epididymus and oviduct respectively [1]. Nevertheless, sperm must regulate their membrane potential (Em) and adapt to these changes in external ion concentration, while also achieving membrane hyperpolarization at appropriate times. We previously showed that the SLO3 sperm-specific, high conductance K^+^ channel was the key channel involved in membrane hyperpolarization during capacitation [2]. However, what was unclear was 1) what is the contribution of SLO3 channels relative to other ion channel types to this hyperpolarization? and 2) what is the overall degree to which SLO3 channels are coupled to other capacitation related processes?

The findings in this manuscript contribute towards the resolution of both of these questions. Regarding the first, two hypotheses were put forward to explain the channel types responsible for sperm Em hyperpolarization: 1) an increase in K^+^ permeability (P_K_), due to the activation of one or more K^+^ selective channels, and 2) a reduction of Na^+^ permeability (P_Na_) by decreasing the activity of Na^+^ channels. The first hypothesis is based on the fact that the hyperpolarization that accompanies mouse sperm capacitation is reduced by K^+^ channel blockers and by increasing the [K^+^]_e_ [3], [4]. In addition, SLO3 high conductance K^+^ channels have now been definitely implicated in capacitation-induced hyperpolarization by our demonstration that sperm from the SLO3 knock-out strain fail to undergo hyperpolarization during capacitation [2]. The second hypothesis which involves the closing of a P_Na_ was raised because of the observations that the sperm membrane potential is hyperpolarized by both a decrease in [Na^+^]_e_, and by the addition of the Na^+^ channel blocker amiloride, both of which produce membrane hyperpolarization in non-capacitated sperm [5]. Based on these facts and immunocytochemical evidence, Hernandez-Gonzalez and co-workers proposed that an epithelial Na^+^ channel is functionally present in mature mouse sperm and the closing of this channel might be at least in part responsible for the hyperpolarization associated with capacitation [5]. It has also been proposed that the trigger that closes ENac is the opening of a CFTR Cl^−^ channel also present in sperm either by direct interaction between these two channels or by Cl^−^ influx [6]. This hypothesis is based on the notion that CFTR inhibitors block the hyperpolarization associated with capacitation, that activation of this channel with genistein produces a hyperpolarization in non-capacitated sperm, and that the CFTR protein is present in the sperm [6]. To address these questions we measured sperm membrane voltage under a variety of conditions and at four different [K^+^]_e_, and fit the data with the Goldman-Hodgkin-Katz equation to reveal the underlying ion permeabilities in each condition. The data presented here shows that P_K_ due to SLO3 activation increases many fold during capacitation, and is by far the most important factor necessary for hyperpolarization; on the other hand, a significant reduction in P_Na_ during capacitation was not noted. Experiments supporting this conclusion are those that show that eliminating the contribution of the SLO3 channel, either by the use of the SLO3 knock-out mutant or by pharmacological agents that block SLO3 P_K_ in wild-type sperm, show no significant hyperpolarization when subjected to capacitating conditions. All experiments revealed that the increase in SLO3 P_K_ is the essential factor in capacitation while significant reduction in P_Na_ does not occur. An additional observation we present is that exposure to external pH 8 both before and after capacitation evokes membrane hyperpolarization in wild-type sperm, but not in SLO3 knock-out mutant sperm or in wild type sperm where pharmacological agents block SLO3 P_K_. Again, in these experiments a significant reduction in P_Na_ does not occur.

Regarding the second question, the capacitation process involves a series of molecular and physiological events that include the activation of a cAMP signaling pathway [7], [8], [9], an increase in intracellular pH [10], [11], the activation of SLO3 channels causing a hyperpolarization of the sperm plasma membrane potential [2], [3], [4], an increase in tyrosine phosphorylation [12], an increase in the intracellular Ca^2+^ concentration ([Ca^2+^]_i_) [13], [14], and a decrease in the [Na^+^]_i_ [5], [15]. However, the sequence of these events, whether they are interrelated or if they take place independently is still not completely understood. Its been proposed that during *in vitro* capacitation pH_i_ is regulated downstream of cAMP synthesis and PKA activation [16]. cAMP might regulate pH_i_ by activation of the sperm specific Na^+^/H^+^ exchanger which has a putative cAMP binding site [17]. Since SLO3 channels are activated by increases in pH_i_, one possible explanation is that intracellular alkalization occurs downstream of a cAMP-dependent pathway and that the increase in pH_i_ then activating SLO3 channels, is responsible for Em hyperpolarization observed during capacitation.

We now show in this manuscript that although this might be the pathway that is operating during *in vitro* capacitation, we can also trigger SLO3-dependent hyperpolarization by increasing external pH independently of other processes that occur during capacitation. We show that external alkalization by itself increases SLO3 permeability by approximately 5 fold, bypassing all other factors detailed above which are part of in-vitro capacitation. These findings suggest that SLO3-dependent hyperpolarization may occur in vivo whenever sperm encounter an alkaline environment during their transit in the female genital tract. In fact alkaline pH values present in the cervix and oviduct especially at the time of ovulation [18] may initiate sperm membrane hyperpolarization prior to other physiological events occurring in capacitation. These results suggest that at least some of the physiological events occurring during capacitation may be independent or at least occur in a different order depending on the conditions encountered by sperm. Moreover, we found that even though SLO3 mutant sperm did not undergo hyperpolarization when incubated under capacitating conditions, other events associated with capacitation do occur, such as a decrease in [Na^+^]_i_ similarly to wild type sperm subjected to capacitating conditions. These results will be discussed with regard to previous reports in the literature.

Our study also revealed a significant membrane permeability to both K^+^ and Cl^−^ before capacitation, as well as Na^+^. We found that the permeability to both K^+^ and Cl^−^ has the effect of preventing large changes in membrane potential when the extracellular concentration of either ion is changed, and may be a factor to stabilize sperm membrane potential prior to capacitation in different ionic environments. Finally our experiments measuring sperm membrane voltage at four different [K^+^]_e_ and fitting the data with the Goldman-Hodgkin-Katz equation have allowed us to make more accurate measurements of ion permeabilities present before capacitation in both wild-type and SLO3 mutant sperm. In our previous experiments we measured sperm membrane voltage in wild-type and SLO3 mutant sperm before capacitation at only one [K^+^]_e_, and we were unable to resolve a significant difference between the two [2]. However by plotting voltages at four different [K^+^]_e_ we now have determined a significant contribution of SLO3 P_K_ in non-capacitated wild-type sperm that is not present in SLO3 mutant sperm.

## Materials and Methods

### Materials

The following materials were purchased from Sigma (St. Louis, MO): Valinomycin (cat. V0627), carbonyl cyanide *m*-chlorophenylhydrazone (CCCP) (cat. C2759), antymicin (cat. A8674), oligomycin (cat. O4876), amiloride (cat. A7410), clofilium (C2365), Barium chloride (B0750), N-Methyl-D-glucamine (cat. 66930), Choline chloride (26980), Sodium methanesulfonate (cat. 304506), Potassium methanesulfonate (cat. 83000) and dimethyl sulfoxide (DMSO) (cat. D8418). 3, 3'-dipropyl- thiadicarbocyanine iodide (DiSC_3_-(5)) (cat. D306) was obtained from Invitrogen (Carlsbad, CA).

### Sperm Preparation

Caudal epididymal sperm were collected from 90 day old C57BL/6 male breeders of either SLO3 mutant or wild-type genotype. SLO3 knock-out mutant mice were generated as previously described [2]. Minced cauda epididymis from each animal were placed in HS medium (in mM): 135 NaCl, 5 KCl, 2 CaCl_2_, 1 MgSO_4_, 20 HEPES, 5 glucose, 10 lactic acid, 1 Na^+^-pyruvate, supplemented with 15 mM of NaHCO_3_ and 5 mg/ml of bovine serum albumin, in pH 7.4 or as indicated in the experiment. For some experiments, we used four different [K^+^]_e_ (in mM): 5, 10, 20 and 30. The swim-up method [19] was used to separate out sperm with >90% motility. The sperm suspension was incubated for 10 min at 37°C, and the top 500 µl, was separated and capacitated by incubating in capacitating medium at 37°C for 60 min. As will be shown, sperm membrane hyperpolarization to a maximum stable level required a 60 min incubation period in the capacitating medium described. In order to compare membrane potential measurements of capacitated sperm with that of non-capacitated sperm in identical media, we made voltage measurements at time 0 of the 60 min incubation period, which we label “non-capacitated sperm” throughout this manuscript.

### Membrane Potential Assay in Cell Populations

A number of fluorescent indicator dyes have been commonly used by several laboratories to evaluate membrane potential. DiSC_3_-(5), which has been successfully used in mammalian sperm [2], [4], [16], [20], is a cationic carbocyanine and thus distributes across cellular membranes in response to electrochemical gradients. Upon hyperpolarization of the plasma membrane the dye enters the cell, binds to intracellular proteins, and undergoes quenching of its fluorescent emission, and exhibits a slight shift in its spectrum with a decrease in its fluorescent signal [21]. This dye has no toxic effects on sperm function at the concentrations used (1 µM) [16]. When constant concentrations of sperm and probe are used, this dye provides reproducible estimates of plasma membrane potential. DiSC_3_-(5) provides a good signal to noise ratio and can be conveniently calibrated using valinomycin. One problem with the use of DiSC_3_-(5) is that this cationic dye binds to mitochondria in their normal energized state, which could conceivably significantly contribute to the fluorescence signal [22]. To determine if this were a source of artifact in our experiments, we performed recordings in the presence and absence of several mitochondrial un-couplers (CCCP, Antimycin and oligomycin) at concentrations indicated in figures S1 and S2. Under all these conditions, the plasma membrane potential of valinomycin-treated mouse sperm responds to the [K^+^]_e_ in accordance with the Nernst equation. We measured the membrane potential at different [K^+^]_e_ using valinomycin to calibrate the signal for each point, and we measured the slopes of this curves. We did not find significant differences in the slopes of the measured curves with any of the mitochondrial un-couplers used (Figures S1 and S2; tables S7 and S8). Hence, we carried out our experiments in the absence of the above-mentioned un-couplers.

Mature sperm from caudal epididymis were capacitated *in vitro* as described above. After incubation at indicated times, the potential sensitive dye DiSC_3_-(5) was added to a final concentration of 1 µM and sperm were incubated for 5 minutes. Incubation for 2 min with CCCP at 500 nM, antimycin at 1 µM or oligomycin at 0.5 µM were used in some control experiments to determine the contribution of mitochondria to the resting potential. After this period, 4×10^6^ sperm were transferred to a gently stirred cuvette at 37°C and the fluorescence was monitored with a Varian Cary Eclipse Fluorescence Spectrophotometer at 620/670 nm excitation/emission wavelength pair [4]. Cell hyperpolarization decreases the dye fluorescence. Recordings were initiated after reaching steady-state fluorescence (1–3 min). Calibration was performed by adding 1 µM valinomycin and sequential additions of KCl [16]. To the initial suspension containing 5 mM KCl, additional KCl was added to final concentrations of (in mM): 7.2, 11.7, 20.7 and 38.7 KCl, corresponding to plasma Em (theoretical) of −84.9, −75.0, −62.1, −46.9 and −30.2 mV, respectively. These values were obtained using the Nernst equation, assuming an [K^+^]_i_ of 120 mM, according to [23]. The final sperm membrane potential was obtained by linearly interpolating the theoretical Em values *versus* arbitrary units of fluorescence of each trace. The fluorescence emission of DiSC_3_-(5) depends on variables such as sperm number, dye concentration, sperm viability, fluorometer cuvette dimensions, and the presence of compounds that interact with DiSC_3_-(5). The internal calibration of each single determination compensates for variables that influence the absolute fluorescence values.

### GHK Curve Fitting

The following assumptions were made regarding GHK curve fitting and the extraction of ionic permeabilities: It was assumed that all ion permeabilities in wild-type and SLO3 mutant non-capacitated sperm would be equal, except for the presence of the ion permeability due to the SLO3 channel in wild-type sperm and its absence in SLO3 mutant sperm. Hence, we began GHK curve fitting with non-capacitated SLO3 mutant sperm, setting the residual P_K_ to 1.0 and solving for optimal P_Cl_ and P_Na_ values. Having established the optimal fit and values of P_Cl_ and P_Na_, we then used these values to fit non-capacitated wild-type sperm. This revealed a higher value of P_K_ in wild-type sperm (P_K_ = 1.6) compared to SLO3 mutant sperm (P_K_ = 1.0). The goodness of fit using these assumptions and derived values could not be significantly improved upon even when all three ion permeabilities were allowed to float.

### Flow Cytometry

Sperm from cauda epididymis from wild type or SLO3 mutant mice were allowed to swim in Whitten’s HEPES-buffered medium for 10 min. These sperm were loaded with 1 µM of CoroNa Red sodium indicator by incubation for 30 min at 37°C in Whitten’s HEPES-buffered medium (in the absence of BSA and HCO_3_^−^). To eliminate non-incorporated dye, sperm suspensions were then centrifuged (5 min, 1000 rpm). Pellets were then resuspended in Whitten’s HEPES-buffered media with or without 15 mM NaHCO_3_^−^ and 5 mg/ml BSA and incubated for a period of 60 min at 37°C. To test the effect of clofilium, sperm were incubated with this compound for 15 min before the centrifugation step. Clofilium was then added back after resuspension. Before FACscan, sperm suspensions were filtered with 100 µm nylon mesh (Small Parts, Inc.) and 2.1 µM of propium iodide (PI) was added. Analyses were conducted using a LSR II flow cytometer (Becton Dickinson, San Jose, CA). CoroNa Red and PI dyes were excited using a 488-nm argon excitation laser. Nonviable cells became PI positive, and their red fluorescent signal detected as fluorescence at a wavelength >670 nm. Orange fluorescence from CoroNa Red positive cells was detected at 561–606 nm. The two dyes have minimal emission overlap. Compensation was done according to guidelines of Roederer (available at http://www.drmr.com/compensation). Non-sperm events were eliminated from analyses by evaluating the events’ scatter properties as detected in the forward-scatter and sideways-scatter detector, (scatter-gated sperm analysis) as described [15]. Recording of scatter and fluorescent properties of all events stopped when 50, 000 events were reached. Two dimensional plots of sideways- and forward-scatter properties (not shown) as well as of PI fluorescence versus CoroNa Red fluorescence were drawn using FlowJo v7.6 software.

### Ethics Statement

It is the policy of Washington University Medical School that all research involving animals be conducted under humane conditions, with appropriate regard for animal welfare. Washington University Medical School is a registered research facility with the United States Department of Agriculture (USDA) and is committed to complying with the Guide for the Care and Use of Laboratory Animals (Department of Health and Human Services), the provisions of the Animal Welfare Act (USDA and all applicable federal and state laws and regulations). At Washington University Medical School an Animal Care Committee has been established to insure compliance with all applicable federal and state regulations for the purchase, transportation, housing and research use of animals. Washington University Medical School has filed appropriate assurance of compliance with the Office for the Protection of Research Risks of the National Institute of Health. All procedures used are designed to ensure avoidance of discomfort, distress or pain. Minimal handling of mice is needed to perform the majority of planned experiments. In all cases the animals will be euthanized before use and the method of euthanasia is outlined below. The described experimental protocol has been approved by the Animal Care and Use Committee at Washington University School of Medicine [Protocol number 2110175]. The method of euthanasia for mice, in all cases, will be CO_2_ asphyxiation followed by rapid cervical dislocation. These methods are consistent with the recommendation of the panel on Euthanasia of the American Veterinary Medical Association and YARC Standard Euthanasia guidelines for rodents.

## Results

### SLO3 K^+^ Channels Contribute to Basal P_K_

**Figure 1:**
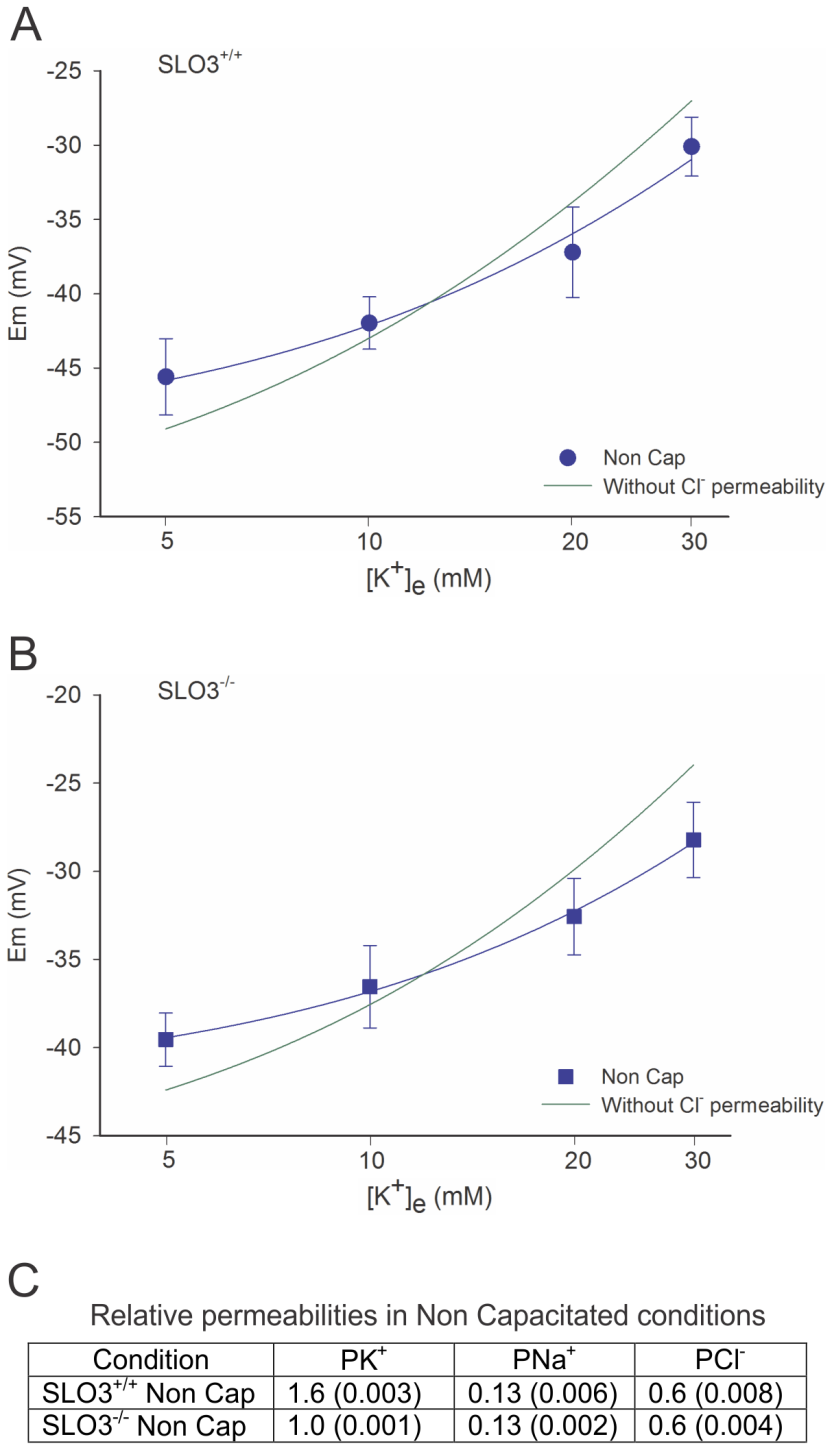
Plots of membrane voltages recorded at different [K^+^]_e_ under non-capacitated conditions. Curves shown are GHK fits to wild-type (A) and SLO3 mutant sperm (B). The green line in both plots is a GHK best fit that omits P_Cl_. Thus, the only way to obtain an accurate fit of the curves with the GHK equation is by including P_Cl_. The relative values of P_K_:/P_Cl_ in non-capacitated sperm are similar to the ones seen in other cell types (see text). All permeabilities given are relative to P_K_ in SLO3 mutant sperm at pH 7.4, which is assigned a value of 1.00. The curves correspond to mean n = 11 experiments. (C) The predicted permeabilities and p values for each permeability in the GHK fit are shown. All measured membrane potential values are given in table S1.

**Figure 2:**
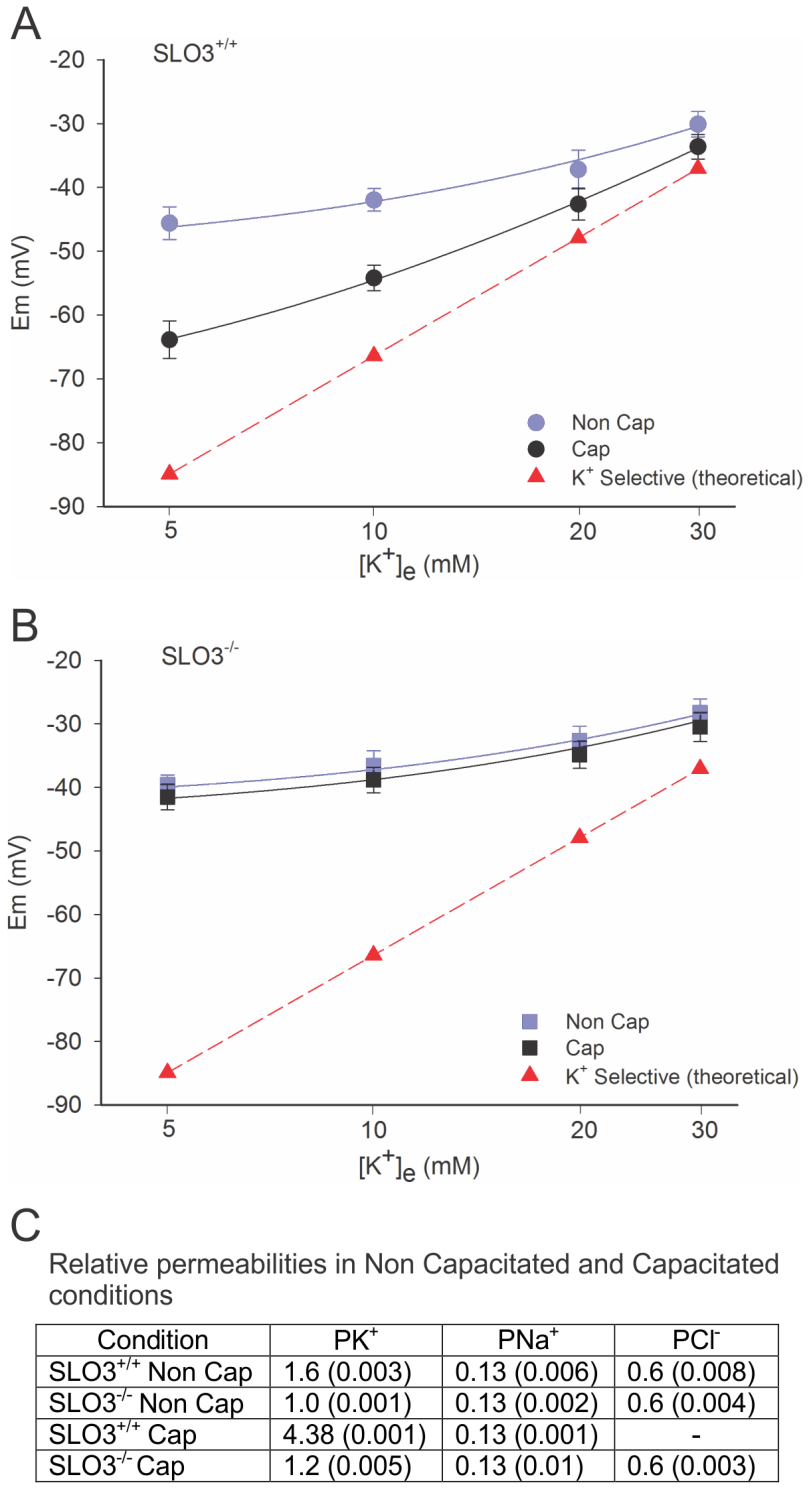
Membrane potential measurements of wild-type and SLO3 mutant sperm in capacitated and non-capacitated conditions. Curves shown are GHK fits to (A) wild-type and (B) SLO3 mutant sperm, prior to and one hour after being subjected to capacitating conditions. The measured membrane potential values seen for capacitated wild-type sperm are likely to represent the average values for a mixed population of sperm (see text). Note that in (B) there is no predicted decrease in P_Na_ in SLO3 mutant sperm subjected to capacitating conditions. The curves correspond to mean n = 11 experiments ± S.E.M. See table S1 for membrane potential values.

**Figure 3:**
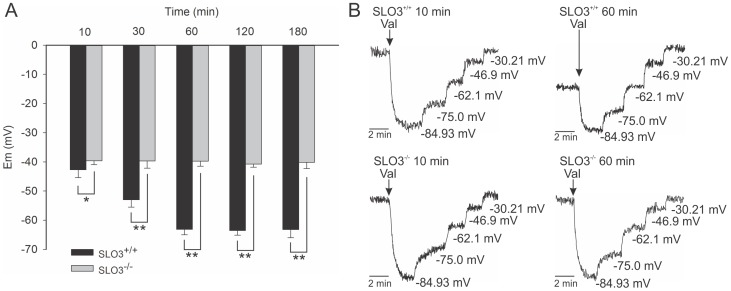
SLO3 mutant sperm do not hyperpolarize even at 180 min in capacitating conditions. Membrane potential measurements (A) and representative plots of florescence emission traces (B) at different capacitation times for wild-type and SLO3 mutant sperm. The bars represent the mean of n = 12 experiments. *indicates (P≤0.05); **indicates (P≤0.01). See table S2 for membrane potential values.

In order to estimate the basal resting cell permeabilities in wild-type and SLO3 mutant sperm, we first made voltage measurements in non-capacitated wild-type and mutant sperm in media containing four different [K^+^]_e_, 5, 10, 20 and 30 mM. The results of these initial measurements revealed that sperm from SLO3 mutant mice were consistently depolarized relative to wild-type under the conditions investigated (pH 7.0, 7.4) (Figures 1, 2 and 3 and table S1). Fitting these results to the Goldman-Hodgkin-Katz (GHK) equation (equation 1) indicated that P_K_ differed between wild-type and SLO3 mutant sperm even in the non-capacitated state; there was a 60% larger P_K_ in wild-type sperm in resting non-capacitated conditions which can be accounted for by a partial activation of SLO3 channels (Figure 1 and 2C; Also see figure S3). We assumed that the P_K_ seen in SLO3 mutant sperm represented the total net P_K_ present in mouse sperm when SLO3 channels were removed. Thus, for purposes of representing relative permeabilities in this manuscript, the basal P_K_ observed in non-capacitated SLO3 mutant sperm at pH 7.4 has been assigned a permeability of 1.0, and all other permeabilities presented in text and tables in this manuscript are relative to this. As will be shown, membrane P_K_ differences between wild-type and SLO3 mutant sperm become much greater at pH 8 and under capacitating conditions.
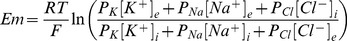
(1)


### Resting Cl^−^ Permeability

As indicated, we found the sperm resting membrane potential prior to capacitation to be ∼−39 to ∼−45 mV (SLO3 mutant and wild-type respectively, at 5 mM external [K^+^]) which is consistent with previous reports, and which is considerably more positive than the estimated K^+^ equilibrium potential (E_K_) (−84.9 mV). As previously suggested, the positive value of the sperm resting membrane potential relative to the estimated E_K_, in all likelihood reflects a contribution from P_Na_ in the resting cell membrane [5]. In addition, the fact that the sperm resting potential is so close to the estimated Cl^−^ equilibrium potential [6] suggests the presence of a significant Cl^−^ permeability (P_Cl_) in the resting membrane. In the process of measuring sperm membrane potential at different [K^+^]_e_ it became obvious that the small positive shifts in resting membrane voltages we observed as [K^+^]_e_ was increased, lagged behind the larger predicted positive shifts in E_K_ (Figure 2). Hence, the membrane potential appeared relatively stable and resistant to depolarization by increases in external K^+^. When fitting the data with the GHK equation it became obvious that this “voltage buffering effect” was due to significant P_Cl_. In Figure 1 the data fitted with GHK equation indicated a relative P_Cl_ of 0.6 relative to the basal P_K_ observed in SLO3 mutant sperm which was assigned 1.0. The steeper green curves shown in figure 1A and 1B show the best GHK fit when P_Cl_ is omitted. Similar experiments and data fits were also undertaken when external Cl^−^ was reduced from 143 to 44 or 5 mM (Figure S4 and tables S9 and S10). As [Cl^−^]_e_ is reduced, the GHK equation predicts that P_Cl_ will have a progressively smaller effect on the resting membrane voltage values even if P_Cl_ values remain the same. In addition, since Cl^−^ concentrations are not maintained by active Cl^−^ ion pumping in sperm cells [24], it is also likely that there will be a redistribution of internal Cl^−^. Thus, when [Cl^−^]_e_ is lowered, the GHK fit of data shows a progressively steeper relation between K_o_ and Em, similar to the green curves shown in Figure 1, and indicating a lower contribution of P_Cl_ in setting the membrane potential (Figure S4). Hence, in normal media, the membrane voltage values obtained at different [K^+^]_e_ could be fit by the GHK equation only if P_Cl_ was taken into consideration, and P_Cl_ was found to be a significant fraction of resting membrane permeability (about half that of P_K_). In non-capacitated sperm, this “dual regulation” of resting membrane potential by both P_K_ and P_Cl_ may be a mechanism for keeping the membrane potential relatively stable in changing ionic environments.

### Ion Permeabilities in Capacitated Wild-type and SLO3 Mutant Sperm

As reported previously [2], and as we show here (Figure 2), we observed membrane hyperpolarization in capacitated wild-type sperm that was not present in SLO3 knock-out mutant sperm. We further observed the absence of hyperpolarization in SLO3 mutant sperm even when subjected to capacitating conditions for 180 min (Figure 3 and table S2). Moreover, we made a special attempt to detect permeability changes in SLO3 mutant sperm subjected to capacitating conditions which would reveal conductances other than that of SLO3 K^+^ channels that may change during capacitation. However, when fitting our voltage data measured at different [K^+^]_e_, we noted that the only change in permeability predicted by the GHK fit was a slight increase in P_K_. The slight increase in P_K_ observed in mutant sperm subjected to capacitating conditions was small but consistently observed. Because of the size of our dataset we were not able to determine whether this slight increase in P_K_ was statistically significant. Nevertheless, a slight P_K_ increase in SLO3 knock-out sperm would be consistent with a previous report that, in the absence of SLO3 channels, a small residual K^+^ conductance retained in SLO3 mutant sperm underwent a slight augmentation when subjected to alkaline pH [25]. It has been suggested that the remaining P_k_ in SLO3 knock-out mice might, at least in part, be CatSper [25]. However, it has been reported that CatSper does not conduct monovalent cations in the presence of 2 mM external Ca^2+^ [26] which is present in all of our experiments. Thus, it is less likely that CatSper is the source of residual K^+^ permeability, but this possibility cannot be completely ruled out.

### Ion Permeabilities in Capacitated Wild-type and SLO3 Mutant Sperm in the Presence of SLO3 Blocking Agents

**Figure 4:**
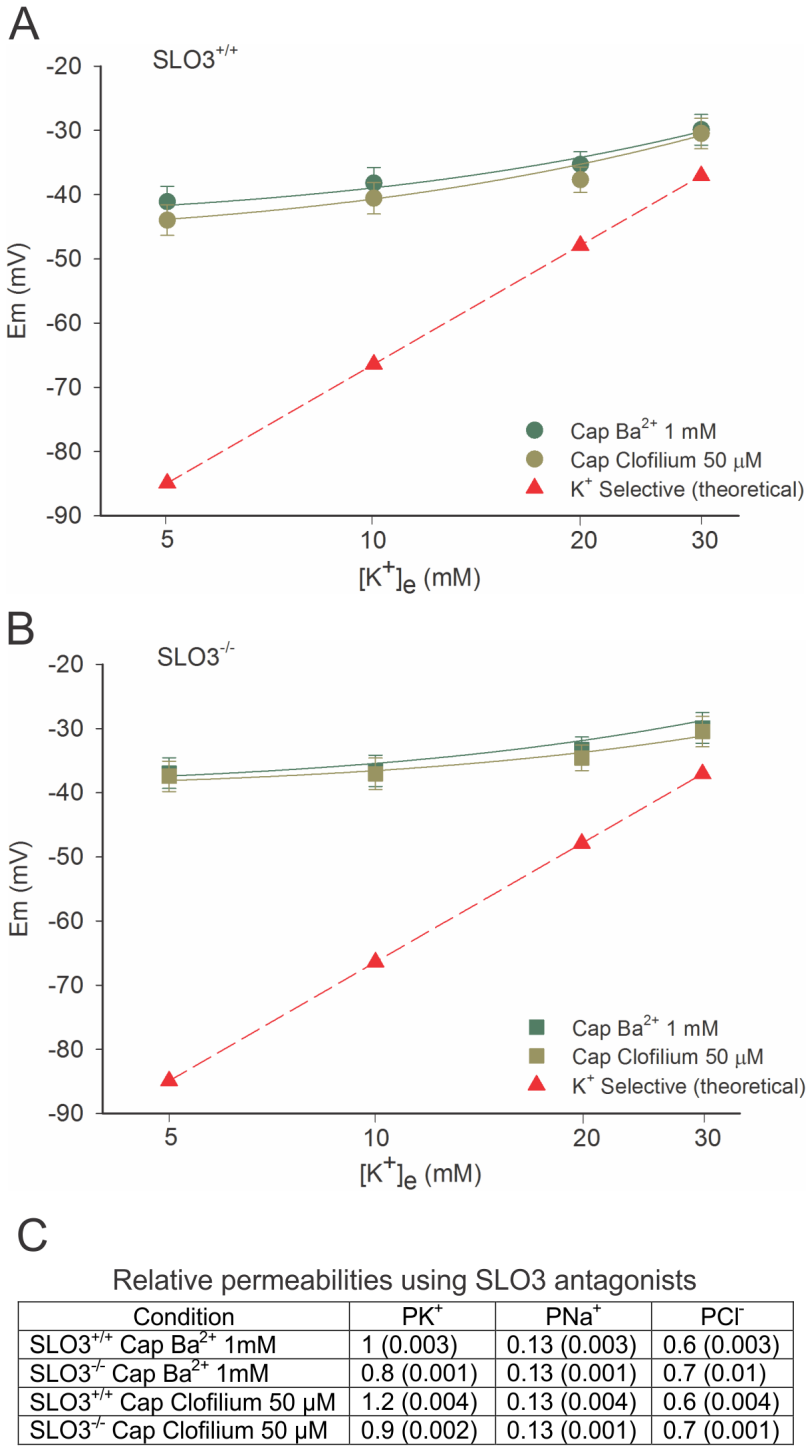
Membrane potential measurements when SLO3 channel antagonists are present in capacitated wild-type and SLO3 mutant sperm; no significant changes in ion permeabilities are seen compared to non-capacitated sperm. Curves shown are GHK fits to wild-type (A) and SLO3 mutant sperm (B). When SLO3 channels are blocked in wild-type sperm, with either Ba^2+^ (1 mM), or clofilium (50 µM), no hyperpolarization is noted during capacitation and membrane permeability values are similar to those of non-capacitated wild-type sperm. Note that there is no predicted decrease in P_Na_ in either wild-type sperm or SLO3 mutant subjected to capacitating conditions. SLO3 blocking agents may have a slight blocking effect on the residual P_K_ in sperm. Permeability values predicted by the GHK equation are given in (C). The curves correspond to mean n = 3 experiments ± S.E.M. See table S3 for membrane potential values.

We also focused on determining membrane permeability changes present in wild-type or SLO3 mutant sperm subjected to capacitating conditions while in the presence of the SLO3 blocking agents 1 mM Ba^2+^ or 50 µM clofilium [27]. This study permitted us to compare relative ion permeabilities in SLO3 mutant sperm (where the SLO3 channel is physically absent), with wild-type sperm, where the SLO3 channel was present, but blocked. The results of this study might reveal membrane permeability changes which were due to developmental defects in forming integrated ion channel complexes in SLO3 mutants. The results of these experiments, however, showed that relative ion permeabilities in wild-type sperm in the presence of SLO3 channel blocking agents are virtually identical to those seen in SLO3 mutant sperm (Figure 4 and table S3). There was also no significant difference between GHK-predicted ion permeabilities seen in SLO3-blocked wild-type sperm prior or after capacitation, except for a tiny increase of P_K_ in capacitated sperm of undetermined statistical significance, as noted in the previous section.

### Ion Permeabilities in Wild-type and SLO3 Mutant Sperm Subjected to Extracellular pH 8 Media before Capacitation

**Figure 5:**
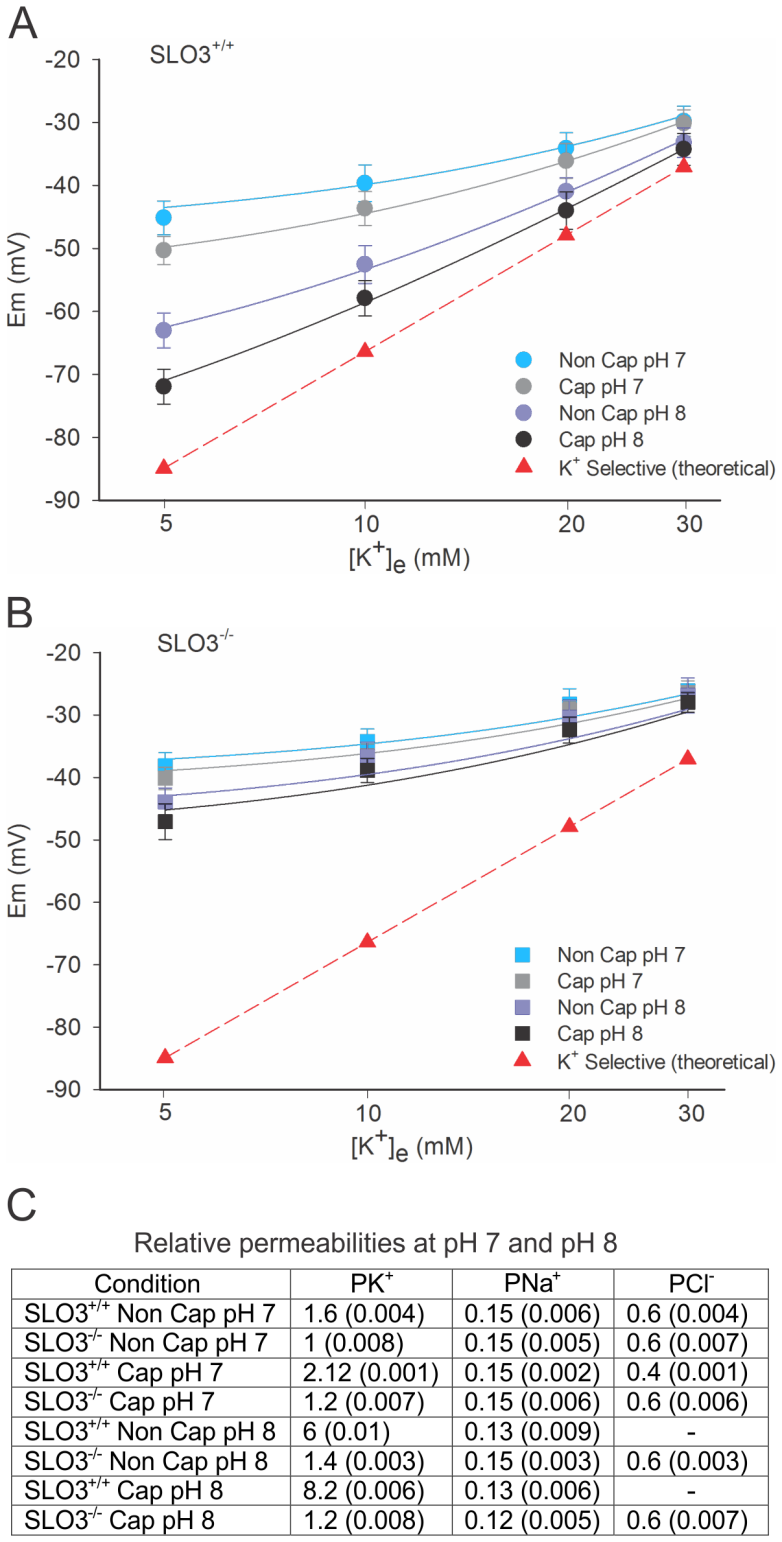
Membrane potential measurements in capacitated and non-capacitated wild-type and SLO3 mutant sperm subjected to extracellular pH 8 media. (A) Wild-type sperm subjected to pH 8 media are hyperpolarized relative to wild-type sperm in pH 7 media; hyperpolarization at pH 8 is evident both before and after capacitation. At pH 7 wild-type sperm subjected to capacitating medium for an hour show a hyperpolarization of only approximately six millivolts. The lack of membrane hyperpolarization at pH 7 may reflect the fact that higher pH media is necessary for the capacitation process [2], [25], [37]. (B) SLO3 mutant sperm subjected to pH 8 media do not hyperpolarize as do wild-type sperm. The slight hyperpolarization present in SLO3 mutant sperm might be due to the pH sensitivity of non-SLO3 potassium permeable channels that remain in SLO3 mutant sperm (see text). Note in (B) that no significant pH 8-dependent hyperpolarization is present which could be attributed to pH-sensitive, non-voltage gated Na^+^ channels, closing at alkaline pH. Permeability values predicted by the GHK equation are given in (C). The curves correspond to mean n = 4 experiments ± S.E.M. See tables S4 and S5 for membrane potential values.

Since it had been reported that sperm intracellular pH approximately follows external pH [10], and since it was discovered that the SLO3 K^+^ channel was activated by alkaline pH [28], we reasoned that SLO3 channels might be activated solely by subjecting sperm to media buffered at alkaline pH. If this were the case then subjecting wild-type sperm to alkaline external media before capacitation should produce significant membrane hyperpolarization, which would be absent in SLO3 mutant sperm similarly treated. Hence we subjected non-capacitated wild-type and SLO3 mutant sperm to pH 8; the results were striking in that wild-type sperm showed extensive hyperpolarization while SLO3 knock-out sperm showed only a small hyperpolarization which might be due to the pH sensitivity of the residual P_K_ (Figure 5 and tables S4 and S5), as previously described [2], [25], or a slight sensitivity of P_Na_ to pH. These results were repeated on both wild-type and SLO3 mutant sperm at pH 8 after being subjected to capacitating conditions, and the results were similar to those observed in sperm subjected to external pH 8 prior to capacitation, except for a statistically insignificant hyperpolarization. The results obtained at pH 8 were in marked contrast to results from wild-type and SLO3 mutant sperm subjected to external pH 7 where very little hyperpolarization was observed even when subjected to capacitating medium for 1 hr. These data suggest: that 1) extracellular alkalization can bypass the capacitation-associated processes that lead to internal alkalization in the activation of SLO3 channels and 2) that intracellular pH regulation is a critical factor in the regulation of hyperpolarization during sperm capacitation. Hyperpolarization in both conditions may result from activation of SLO3 channels by raising intracellular pH. These results show that SLO3-dependent hyperpolarization can be achieved by an alkaline environment alone independently to other associated capacitation processes.

It is worth emphasizing that, unlike wild-type sperm, subjecting SLO3 mutant sperm to pH 8 produces only a small membrane hyperpolarization (Figure 5, B and table S5). This slight hyperpolarization observed in SLO3 mutant sperm from going from pH 7 to 8 may be due to a slight decrease in sodium ion permeability after capacitation. A small decline in P_Na_ after alkalization might be expected since amiloride-sensitive Na^+^ channels have been reported to be sensitive to external pH [29]. This result shows the primacy of SLO3 K^+^ channels in pH-dependent hyperpolarization at an elevated pH. Thus, the permeability changes shown in Figure 5 at pH_o_ 8 for wild-type sperm show that the activation of SLO3 channels accounts for approximately 95% of membrane potential changes, where P_K_ increases approximately 5 times (from a relative P of 1.6 to 8.2). Spermatozoa of SLO3 mutant mice undergo only about 5% of the hyperpolarization seen in wild-type which, as discussed, may be due to a slight increase in the P_K_ of other K^+^ channels (from 1 to 1.2) and conceivably a slight decrease in P_Na_ (from 0.15 to 0.12).

Additionally, it should be noted that to achieve the higher levels of hyperpolarization measured, the GHK equation predicts that either P_Cl_ must decrease, or the internal [Cl^−^] must decrease (to approximately 10–12 mM). The first possibility would result if P_Cl_ were voltage sensitive, perhaps if due to an outward rectifying CLC-type Cl^−^ channel. The second possibility would result if the secondary methods of controlling [Cl^−^] present in sperm [24] were overwhelmed when a major hyperpolarizing mechanism were activated in sperm, such as a large increase in P_K_ due to the activation of high conductance SLO3 channels. As membrane hyperpolarization occurred, Cl^−^ would be driven outward until new equilibrium conditions ensued. Redistribution of Cl^−^ would be expected to be less at the higher [K^+^]_e_ where E_K_ and E_Cl_ approach convergence at normal (non-capacitated, pH 7.4) conditions. The same considerations regarding a reduction in P_Cl_, or Cl^−^ redistribution must also be taken into consideration in the following section where we show that membrane hyperpolarization is achieved by blocking P_Na_ with amiloride, or by reducing the [Na^+^]_e_.

### Ion Permeabilities in Wild-type and SLO3 Mutant Sperm with Amiloride-blocked P_Na_

**Figure 6:**
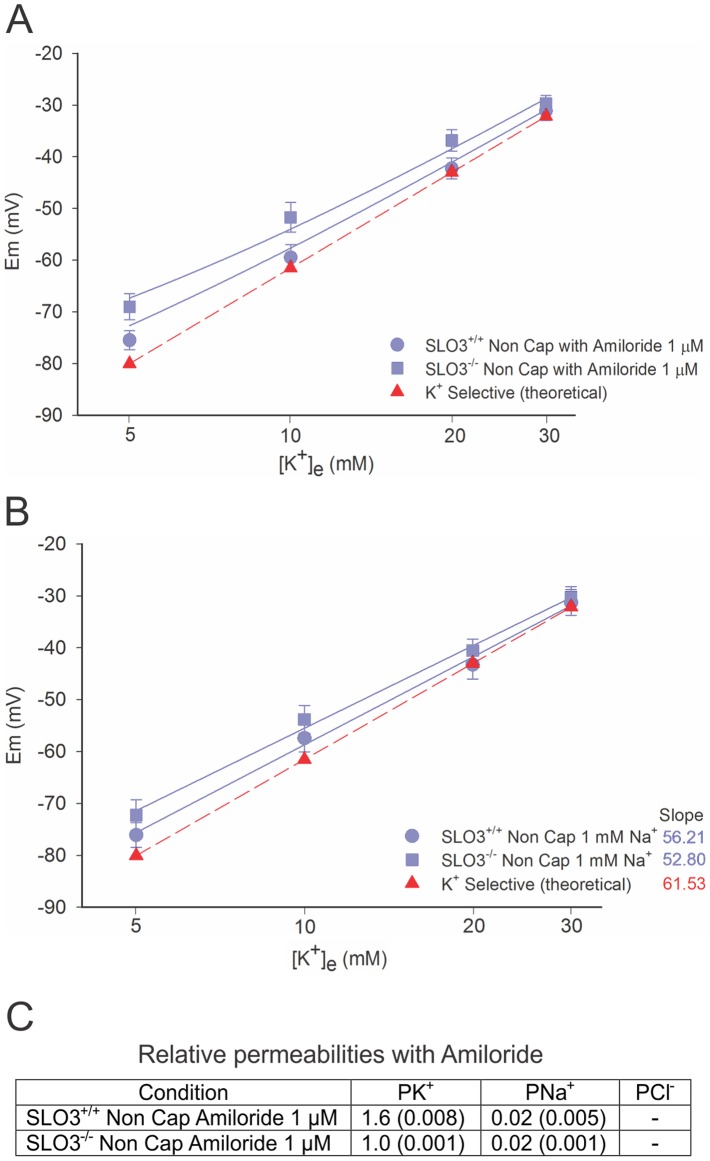
Amiloride or the reduction of external Na^+^ blocks most P_Na_, leaving the membrane dominated by P_K_. Amiloride treatment (A) and low external Na^+^ (B) leave the sperm membrane dominated by P_K_ in both wild-type and in SLO3 mutant sperm. After the addition of amiloride the P_K_ to P_Na_ ratio is somewhat larger in wild-type than in the SLO3 mutant, reflecting the activity of SLO3 K^+^ channels in the membrane (see text). Although the SLO3 channel is absent in SLO3 mutant sperm, the dominance of P_K_ over all other ion permeabilities in SLO3 mutant sperm is additional evidence for the presence of a K^+^ leak conductance in SLO3 mutant sperm plasma membrane. GHK fits did not require inclusion of P_Cl_ (see text). Since we cannot accurately predict the internal sodium concentration when external Na^+^ is reduced to 1 mM, figure 6B is fitted with least squares linear regression to compare the resulting slopes with that of a theoretical line illustrating pure potassium selectivity (red). Permeability values predicted by the GHK equation for A are given in (C). The curves correspond to mean n = 4 experiments ± S.E.M. See table S6 for membrane potential values.

As previously reported [5], [30], membrane hyperpolarization is also achieved when amiloride is added, or media containing low [Na^+^]_e_ (1 mM) is used in non-capacitated sperm. As in the previously presented experiments, we undertook these experiments at different [K^+^]_e_ to get a better estimate of the remaining membrane ion permeabilities after each treatment. Figure 6 shows that fitted curves were steeper and straighter as would be predicted when membrane permeability was more heavily dominated by a single ion species. Amiloride (Figure 6A) appears to reduce the P_Na_ by a factor of 6.5, leaving the membrane largely K^+^ selective; this is true even in SLO3 mutant sperm, where the P_K_ to P_Na_ ratio changes from ∼8∶1 before amiloride treatment to ∼50∶1 after amiloride treatment. In wild-type sperm, the P_K_ to P_Na_ ratio changed from ∼12∶1 before amiloride treatment to ∼80∶1 after amiloride treatment (Figure 6). This is reflected in the fact that, the GHK equation predicts a nearly straight line for the data in Figure 6 (see also table S6 for membrane potential values). Indeed, when the data for each experiment shown in Figure 6B is fitted with a straight line the slope of each line approaches that of the theoretical line illustrating a purely K^+^ selective membrane.

### SLO3-dependent Membrane Hyperpolarization is Independent of the Capacitation-associated Decrease in [Na^+^]_i_

**Figure 7:**
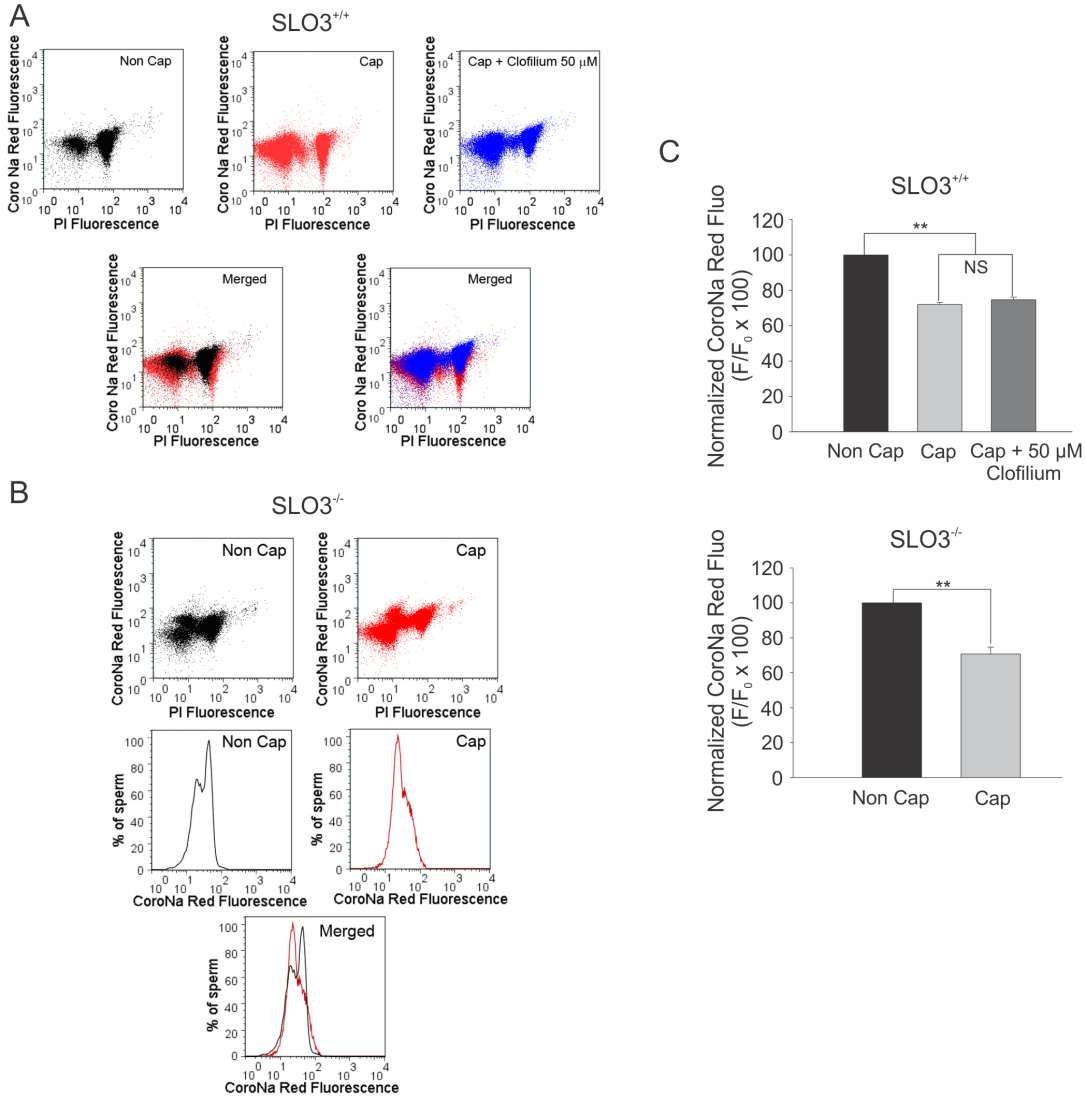
[Na^+^]_i_ is decreased in SLO3 mutant sperm and in sperm treated with the SLO3 inhibitor clofilium. Cauda epididymal sperm from wild type or SLO3 mutant mice were recovered and loaded with CoroNaRed in media lacking BSA and HCO_3_^−^ which does not support capacitation (Non Cap). After thirty minutes incubation, the sperm were washed once and resuspended in the same media or in media containing BSA and HCO_3_^−^ (Cap) in the absence or in the presence of clofilium (50 µM) (for wild-type sperm). After 1 hour incubation, PI was added and the sperm population analyzed by flow cytometry. A) SLO3 wild-type sperm: PI vs CoroNa Red two-dimensional dot plots of sperm incubated in non capacitating conditions (Non Cap), in media that support capacitation (Cap) or in media that support capacitation in the presence of clofilium (Cap+Clofilium 50 µM). The left merged panel combined data from Non Cap and Cap dot plots, the right merged panel combined the Cap and the Cap+clofilium dot plots. B) SLO3 mutant sperm: PI vs CoroNa Red two-dimensional dot plots of sperm incubated in non capacitating conditions (Non Cap) or in media that support capacitation (Cap). Live sperm populations in each case were then analyzed for their individual [Na^+^]_i_ CoroNa Red fluorescence histograms (Non Cap and Cap); the merged panel combined both data. C) Summary for plots showed in A and B. The bars represent the mean n = 3 experiments ± S.E.M. NS indicates No statistical significance (P≥0.05), **indicates statistical significance (P≤0.01).

Several functional changes occur during sperm capacitation including the activation of a cAMP signaling pathway [7], [8], [9], an increase in intracellular pH [10], [11], an increase in tyrosine phosphorylation [12], an increase in [Ca^2+^]_i_ [13], [14], and a decrease in [Na^+^]_i_ [5], [15]. The availability of the SLO3 K^+^ channel knockout has given us a tool to investigate how tightly coupled these events are to the SLO3-dependent hyperpolarization of the sperm plasma membrane potential. We found that PKA and tyrosine phosphorylation also occur in SLO3 mutant sperm (data not shown), indicating that some capacitation-associated molecular and physiological changes are either upstream or are independent of SLO3-elicited membrane hyperpolarization. In particular, SLO3 mutant sperm as well as wild-type sperm in the presence of the SLO3 inhibitor clofilium, both undergo a decrease in [Na^+^]_i_ (Figure 7). On the other hand, in the current study we show that wild-type sperm can undergo SLO3-dependent hyperpolarization in non-capacitating conditions when exposed to an alkaline medium, showing the events occurring during sperm membrane capacitation are either upstream or independent of membrane hyperpolarization. When sperm are subjected to *in vitro* capacitating conditions at pH 7.4, hyperpolarization is blocked by PKA inhibitors; alternatively, non-capacitated sperm undergo hyperpolarization when incubated with cAMP agonists [16]. These data suggest that under those conditions, intracellular alkalinization and consequently SLO3 activation are downstream PKA activation.

## Discussion

### A more Complete Picture of Ion Permeabilities Prior to and after Capacitation

We measured the sperm cell membrane potential at different [K^+^]_e_ and under a variety of conditions and fitted our data with the GHK equation to estimate the different ion permeabilities and how these permeabilities change as a consequence of the capacitation process. We found that non-capacitated sperm submitted to different [K^+^]_e_ have a membrane potential that is governed not only by the interplay of a P_K_ and P_Na_ but also by a P_Cl_ which we estimated to be a significant fraction of resting membrane permeability. Importantly, the resting potential of non-capacitated sperm is close to the equilibrium potential for Cl^−^ ion. As reported before [3], [4], [6], we noted that in non capacitating conditions, raising the external [K^+^] or lowering the external [Cl^−^] elicits a membrane depolarization which is less than the predicted positive shift of the respective ion equilibrium potentials. Thus, the membrane potential in non-capacitated sperm appears to be “buffered” against depolarization when confronted with different external ionic conditions. Mammalian sperm encounter environments with very different ionic composition on their journey to meet the egg. For example [K^+^]_e_ may change from ∼39 to 5–8 mM, [Cl^−^]_e_ from ∼27 to 130 mM, and [Na^+^]_e_ from 38 to 140 mM in the cauda epididymis and oviduct respectively [1]. Nevertheless, sperm must regulate their membrane potential and adapt to these changes in external ion concentration. Our data suggests that the relative membrane potential stability of non-capacitated sperm can be explained by a significant P_Cl_ as well as P_K._The relative permeabilities between K^+^ and Cl^−^ measured in sperm are similar to those seen in the literature. For example, the P_K_ to P_Cl_ ratio measured in crayfish neurons varied between 1.0∶0.26 to 1.0∶0.63 depending on the [K^+^]_e_ [31]. These values are similar to our estimates suggesting an approximate P_K_ to P_Cl_ ratio of 1.0∶0.37 for wild-type and 1.0∶0.6 for SLO3 mutant sperm, in the resting non-capacitated state (Figures 1 and 2). Several factors should be considered in our experiments which may introduce errors. As in the Strickholm study [31], the relative P_K_ to P_Cl_ ratio may change at the different [K^+^]_e_ studied. However our current study is conducted over a more narrow range of [K^+^]_e_. Additionally, sperm may undergo ion redistributions when subjected to different conditions. Nevertheless our results are highly consistent and data curves are well fit by the GHK equation with the permeability values presented (Figure 2). The one relative permeability factor that differs most in sperm cells relative to that of neurons is the lower estimated resting cell P_K_ to P_Na_ ratio which is approximately 1.0∶0.13 = 7.7 for SLO3 mutant sperm, and 1.6∶0.13 = 12 for wild-type. In neurons at rest, this ratio is commonly greater than 30, and the lower value estimated in sperm is reflected in a relatively more positive membrane potential than is found in most neurons. As showed, this relatively high P_Na_ is a consequence of an amiloride-sensitive Na^+^ channel present in the membrane which shows no apparent rectification with voltage. The fact that sperm cells have a resting membrane potential close to E_Cl_ and distant from E_K_ suggests that, unlike some neurons, Cl^−^ concentrations are regulated by indirect means rather than by an active ATP-driven pump. Although there are several mechanisms for regulating Cl^−^ distribution in sperm and thus, Cl^−^ is not entirely passively distributed [24], our data suggests that, when subject to a strong hyperpolarizing influence such as the block of P_Na_ by amiloride, Cl^−^ ions tend to move until E_Cl_ is close to the membrane resting potential. However, the GHK fits to our data suggest that in non-capacitated sperm, [Cl^−^]_i_ remains relatively stable as the external [K^+^] is changed (Figure 2). In non-capacitated sperm, this “dual regulation” of resting membrane potential by both P_K_ and P_Cl_ may be a mechanism for keeping the membrane potential relatively stable in changing ionic environments. This study of the permeabilities present in non-capacitated sperm also revealed that SLO3 high conductance K^+^ channels also contribute to membrane resting potential prior to capacitation.

### Role of the SLO3 Channel

It is clear from many experiments that the pH-dependent activation of SLO3 K^+^ channels is the essential element of sperm membrane hyperpolarization. The SLO3 K^+^ channel is a member of the high conductance SLO K^+^ channel family and was cloned in 1998 in the Salkoff lab [28]. SLO3 channels are only present in mammalian sperm and are activated by intracellular alkalization and membrane depolarization [28], [32], [33]. SLO3 channels are fairly resistant to block by external TEA (a typical K^+^ channel blocker), but are very sensitive to low concentrations of external Ba^2+^, clofilium and quinidine [34], [35]. The sperm K^+^ current called IKSper, identified in corpus epididymal sperm has been shown to be carried by SLO3 channels [25], [27]. The ion specificity of SLO3 channels for K^+^ over Na^+^ may not be as high as that for some other K^+^ channels [28] but our current study does not address that question. In no instance we were able to create conditions where the membrane was exclusively permeable to K^+^ ion; even when amiloride was added, the sperm membrane retained some P_Na_. In addition, in wild-type sperm there is always a contribution from non-SLO3 K^+^ channels present in the membrane.

It has been reported that in sperm samples subjected to capacitating medium, only 40% or less actually achieve the capacitated state, resulting in a heterogeneous sperm population [3], [36]. Furthermore, it has been determined that the sperm cells in such a heterogeneous population have resting membrane potentials that fall into two subpopulations; those that appear not to have achieved a capacitated state with a membrane potential less than −50 mV, while those that appear to have achieved capacitation with membrane potentials approaching −80 mV [36]. Therefore, it is likely that at pH 7.4 in wild-type, the membrane potential results we report for the capacitated state actually correspond to a mix of two subpopulations, and the values we obtained correspond to the average of these populations. On the other hand, at pH 8 under capacitating conditions, it appears that we have a more uniform population where the majority of cells may have achieved hyperpolarization. Thus, the calculated P_K_ of approximately 4 for wild-type sperm in the capacitated state at pH 7.4, is approximately half that of the calculated P_K_ of approximately 8 for wild-type sperm in the capacitated state at pH 8.0, which could reflect a mixed population of sperm at pH 7.4.

### Sodium Permeability in Sperm Plama Membrane

In addition to the pH-dependent increase in P_K_ contributed by SLO3 channels during capacitation, a second potential mechanism to achieve membrane hyperpolarization involved the lowering of P_Na_. This premise is partially based on the fact that sperm resting membrane potential prior to capacitation has been measured to be more positive than the K^+^ equilibrium potential suggesting the participation of other ions in setting the sperm membrane potential [3], [4], [16], [20], [36]. It was also bolstered by the fact that the membrane potential in non-capacitated sperm is hyperpolarized either by a decrease in [Na^+^]_e_, or by the addition of the (non-voltage-dependent) Na^+^ channel blocker, amiloride [5]. Based on these facts and immunocytochemical evidence, Hernandez-Gonzalez et al, proposed that an epithelial Na^+^ channel is functionally present in mature mouse sperm and the closing of this channel might be at least in part responsible for the hyperpolarization associated with capacitation [5]. However, our experiments only indicate a slight decrease in P_Na_ in either wild-type or SLO3 mutant sperm either after capacitation, or prior to capacitation when hyperpolarization is elicited by pH 8 external media. Although, previous results suggested that the closing of Na^+^ channels after capacitation might have a larger influence on hyperpolarization, our current results obtained using SLO3 mutant sperm strongly suggest that the physiological hyperpolarization induced under capacitating conditions is not dependent on reduction of Na^+^ permeability but on a very large increase in SLO3-dependent P_K_ seen after capacitation. Indeed our current results present an explanation for previous results that were interpreted as a decrease in P_Na_ after capacitation [5]. In those studies it was shown that increasing the [Na^+^]_e_ (in a low sodium media) before or after capacitation produced markedly different degrees of depolarization; before capacitation added Na^+^ produced a larger depolarization. This was interpreted as a lowering of P_Na_ after capacitation. However, our current results showing relative P_K_/P_Na_ before and after capacitation suggest a different interpretation: Before capacitation we observe a P_K_/P_Na_ ration of 1.6/.13 = 12.3; after capacitation we observe a P_K_/P_Na_ ratio of 4.38/.13 = 33. Substituting these values into the GHK equation shows that adding external Na^+^ after capacitation produces a much lower change in voltage than does the addition of external Na^+^ before capacitation, and that this difference is due to the large increase in P_K_ after capacitation, rather than a decrease in P_Na_.

### How Tightly Coupled is Membrane Hyperpolarization to other Capacitation-associated Processes?

It is clear that the main factor that activates SLO3 channels producing a hyperpolarization of the sperm plasma membrane is the increase in pH_i_. However, how intracellular pH is regulated is a matter of speculation at present. It is likely that during *in vitro* capacitation, pH_i_ is regulated downstream of cAMP synthesis and PKA activation [16]. In this regard, a sperm specific Na^+^/H^+^ exchanger has a cAMP-binding domain suggesting that it can be regulated by cAMP binding [17]. Also, we have shown that the addition of cAMP agonists can induce hyperpolarization in conditions that do not support capacitation (absence of HCO_3_^−^), and that the addition of H89, a PKA inhibitor, blocks the capacitation-associated hyperpolarization [16]. These results taken together appear to suggest that intracellular alkalization is downstream of a cAMP-dependent pathway and that the increase in pH_i_ is responsible for Em hyperpolarization observed during *in vitro* capacitation. On the other hand we also found that PKA and tyrosine phosphorylation occur in SLO3 mutant sperm (data not shown), indicative of the possibility that some of these capacitation-associated molecular and physiological changes are either independent or upstream of SLO3-elicited membrane hyperpolarization.

### Responsiveness of Sperm Membrane Potential to External Alkaline pH

Our results showing the responsiveness of sperm membrane potential to external alkaline pH suggest the possibility that sperm membrane potential changes may occur at various stages of a sperm’s travel to the egg independently of other capacitation associated processes. Since it has been shown that pH_i_ will increase by following an increase in external pH [10], and that an increase in pH_i_ alone is sufficient to activate SLO3 channels [28], it follows that a rise in external pH alone is sufficient to activate SLO3 channels. Thus in vivo, hyperpolarization may occur whenever the sperm encounter an alkaline environment independently to other capacitation associated processes. It follows then that hyperpolarization will be expected to occur when sperm enter the alkaline environment of the cervix and the oviduct [18]. These results suggest that the ability of mouse sperm to sense external alkaline pH and react with membrane hyperpolarization is almost entirely dependent on SLO3 channels.

## Supporting Information

Figure S1 **Plots of membrane potential measurements compared between experiments with and without the mitochondrial un-coupler CCCP, in non-capacitated and capacitated conditions.** Curves shown are GHK fits to wild-type (A) and SLO3 mutant (B) measured voltages, with and without CCCP. No significant differences (P≥0.05) were found between corresponding membrane voltage values measured with or without CCCP, either for wild-type or mutant data. Permeability values predicted by the GHK equation are given in (C). The curves correspond to mean n = 5 experiments ± S.E.M. See table S7 for membrane potential values.
(TIF)

Figure S2 **Plots of membrane potential measurements with the mitochondrial un-couplers Antimycin and Oligomycin, in non-capacitated and capacitated conditions.** Curves represent GHK fits to wild-type (A) and SLO3 mutant (B) in the presence of both antimycin and oligomycin. These data were compared to control experiments without these mitochondrial un-couplers, and no significant differences (P≥0.05) were found. Permeability values predicted by the GHK equation are given in (C). The curves correspond to mean n = 3 experiments ± S.E.M. See table S8 for membrane potential values.
(TIF)

Figure S3 **Membrane potential measurements of wild-type and SLO3 mutant sperm in non-capacitated conditions.** Curves shown are GHK fits comparing wild-type and SLO3 mutant sperm in non-capacitating conditions. The curves reveal that SLO3 mutant sperm are more depolarized than wild-type in non-capacitating conditions (See text), and this difference is statistically significant (P≤0.05) (to compare the curves we used chi-square test). The curves correspond to mean n = 11 experiments ± S.E.M. See table S1 for membrane potential values.
(TIF)

Figure S4 **Plots of membrane potential measurements in low [Cl^−^]_e_ in non-capacitated and capacitated conditions in wild-type sperm.** Curves represent GHK fits in wild-type sperm in 44 mM (A) and 5 mM (B) external Cl**^−^**. These curves are steeper than those shown in figure 1 where the voltage measurements were made under conditions of normal [Cl**^−^**]_e_. Permeability values predicted by the GHK equation are given in (C). Unlike figure 1, the GHK fits did not require consideration of chloride ion permeability (see text). The curves correspond to mean n = 3 experiments ± S.E.M. See tables S9 and S10 for membrane potential values.
(TIF)

Table S1 **Membrane potentials in Non capacitated and Capacitated conditions.** Em values obtained at the indicated external K^+^ concentrations, in wild-type (SLO3^+/+^) or SLO3 mutant (SLO3**^−^** ^/**−**^) sperm under Non capacitated (Non Cap) and Capacitated (Cap) conditions. Values are given in millivolts (mV) and correspond to mean n = 11 and numbers within brackets correspond to S.E.M.
(DOC)

Table S2 **Membrane potentials during different time of capacitation.** Em values obtained at the indicated incubation times, in wild-type (SLO3^+/+^) or SLO3 mutant (SLO3**^−^** ^/**−**^) sperm. Values are given in millivolts (mV) and correspond to mean n = 12 and numbers within brackets correspond to S.E.M.
(DOC)

Table S3 **Membrane potentials using SLO3 antagonists.** Em values obtained at the indicated external K^+^ concentrations, in wild-type (SLO3^+/+^) or SLO3 mutant (SLO3**^−^** ^/**−**^) sperm under Capacitated (Cap) conditions in the presence of Ba^2+^1 mM and Clofilium 50 µM. Values are given in millivolts (mV) and correspond to mean n = 3 and numbers within brackets are S.E.M.
(DOC)

Table S4 **Membrane potentials in pH 7.** Em values obtained at the indicated external K^+^ concentrations, in wild-type (SLO3^+/+^) or SLO3 mutant (SLO3**^−^** ^/**−**^) sperm under Non capacitated (Non Cap) and Capacitated (Cap) conditions in external pH 7. Values are given in millivolts (mV) and correspond to mean n = 4 and numbers within brackets correspond to S.E.M.
(DOC)

Table S5 **Membrane potentials in pH 8.** Em values obtained at the indicated external K^+^ concentrations, in wild-type (SLO3^+/+^) or SLO3 mutant (SLO3**^−^** ^/**−**^) sperm under Non capacitated (Non Cap) and Capacitated (Cap) conditions in external pH 8. Values are given in millivolts (mV) and correspond to mean n = 4 and numbers within brackets correspond to S.E.M.
(DOC)

Table S6 **Membrane potentials with Amiloride and low [Na^+^]_e_.** Em values obtained at the indicated external K^+^ concentrations, in wild-type (SLO3^+/+^) or SLO3 mutant (SLO3**^−^** ^/**−**^) sperm under Non capacitated (Non Cap) and Capacitated (Cap) conditions in the presence of Amiloride 1 µM or in 1 mM external Na^+^. Values are given in millivolts (mV) and correspond to mean n = 4 and numbers within brackets correspond to S.E.M.
(DOC)

Table S7 **Membrane potentials using mitochondrial un-coupler CCCP.** Em values obtained at the indicated external K^+^ concentrations, in wild-type (SLO3^+/+^) or SLO3 mutant (SLO3**^−^** ^/**−**^) sperm under Non capacitated (Non Cap) and Capacitated (Cap) conditions in the presence of CCCP 0.5 µM. Values are given in millivolts (mV) and correspond to mean n = 5 and numbers within brackets correspond to S.E.M.
(DOC)

Table S8 **Membrane potentials** **using mitochondrial un-couplers Antimycin and Oligomycin.** Em values obtained at the indicated external K^+^ concentrations, in wild-type (SLO3^+/+^) or SLO3 mutant (SLO3**^−^** ^/**−**^) sperm under Non capacitated (Non Cap) and Capacitated (Cap) conditions in the presence of Antimycin 1 µM and Oligomycin 0.5 µM. Values are given in millivolts (mV) and correspond to mean n = 3 and numbers within brackets correspond to S.E.M.
(DOC)

Table S9 **Membrane potentials in SLO3^+/+^ using 44 mM Cl^−^ external.** Em values obtained at the indicated external K^+^ concentrations, in wild-type (SLO3^+/+^) sperm under Non capacitated (Non Cap) and Capacitated (Cap) conditions using 44 mM external Cl^−^. Values are given in millivolts (mV) and correspond to mean n = 3 and numbers within brackets correspond to S.E.M.
(DOC)

Table S10 **Membrane potentials in SLO3^+/+^ using 5 mM Cl^−^ external.** Em values obtained at the indicated external K^+^ concentrations, in wild-type (SLO3^+/+^) sperm under Non capacitated (Non Cap) and Capacitated (Cap) conditions using 5 mM external Cl^−^. Values are given in millivolts (mV) and correspond to mean n = 3 and numbers within brackets correspond to S.E.M.
(DOC)
