# Supplementary figures and images for: Ion Permeabilities in Mouse Sperm Reveal an External Trigger for SLO3-Dependent Hyperpolarization
Source: PLoS One. 2013 Apr 5;8(4):e60578. doi: 10.1371/journal.pone.0060578 (PMC3618424; doi:10.1371/journal.pone.0060578)

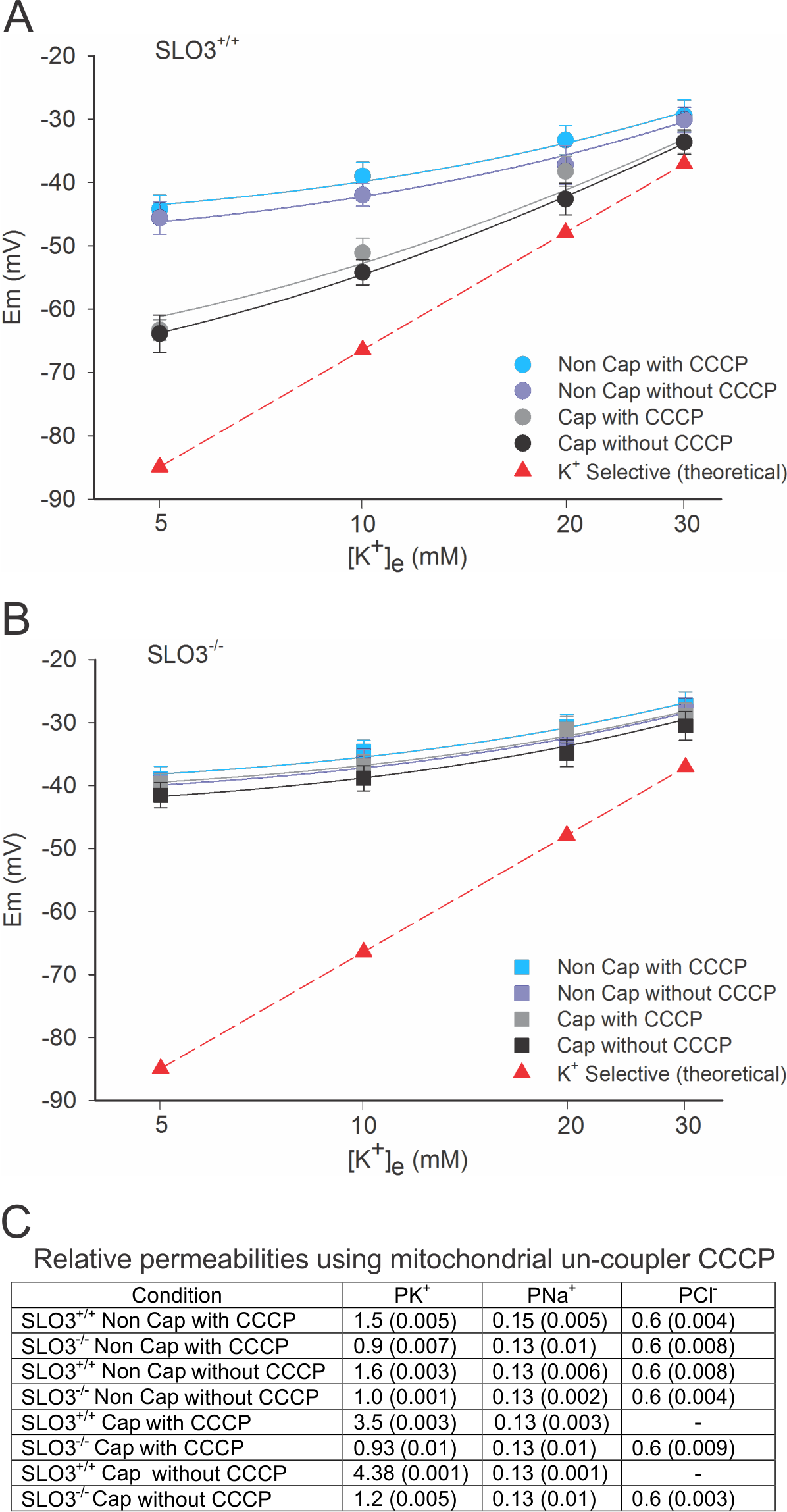

Supplement: Figure S1 — Plots of membrane potential measurements compared between experiments with and without the mitochondrial un-coupler CCCP, in non-capacitated and capacitated conditions. Curves shown are GHK fits to wild-type (A) and SLO3 mutant (B) measured voltages, with and without CCCP. No significant differences (P≥0.05) were found between corresponding membrane voltage values measured with or without CCCP, either for wild-type or mutant data. Permeability values predicted by the GHK equation are given in (C). The curves correspond to mean n = 5 experiments ± S.E.M. See table S7 for membrane potential values. (TIF) [file pone.0060578.s001.tif]

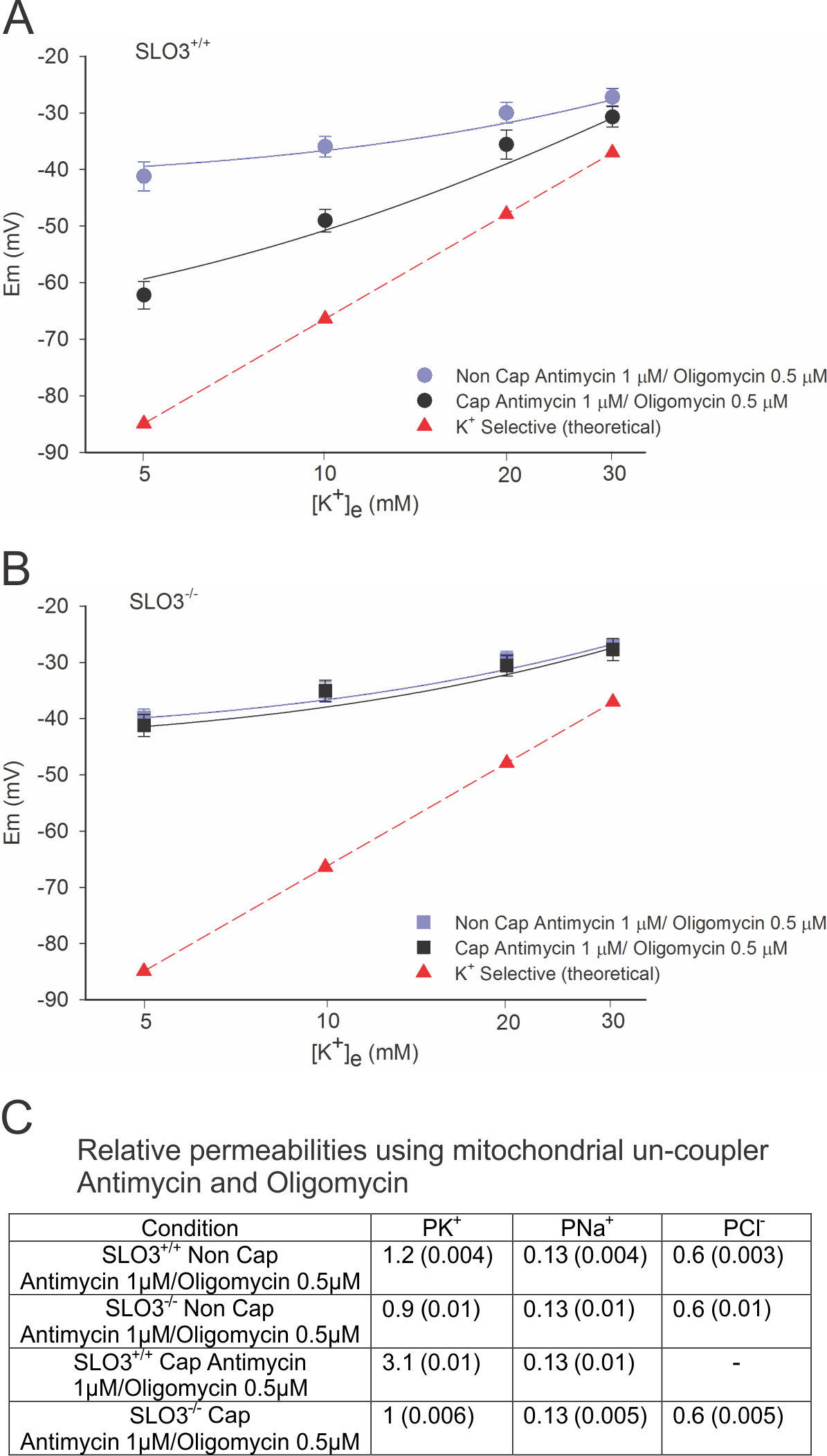

Supplement: Figure S2 — Plots of membrane potential measurements with the mitochondrial un-couplers Antimycin and Oligomycin, in non-capacitated and capacitated conditions. Curves represent GHK fits to wild-type (A) and SLO3 mutant (B) in the presence of both antimycin and oligomycin. These data were compared to control experiments without these mitochondrial un-couplers, and no significant differences (P≥0.05) were found. Permeability values predicted by the GHK equation are given in (C). The curves correspond to mean n = 3 experiments ± S.E.M. See table S8 for membrane potential values. (TIF) [file pone.0060578.s002.tif]

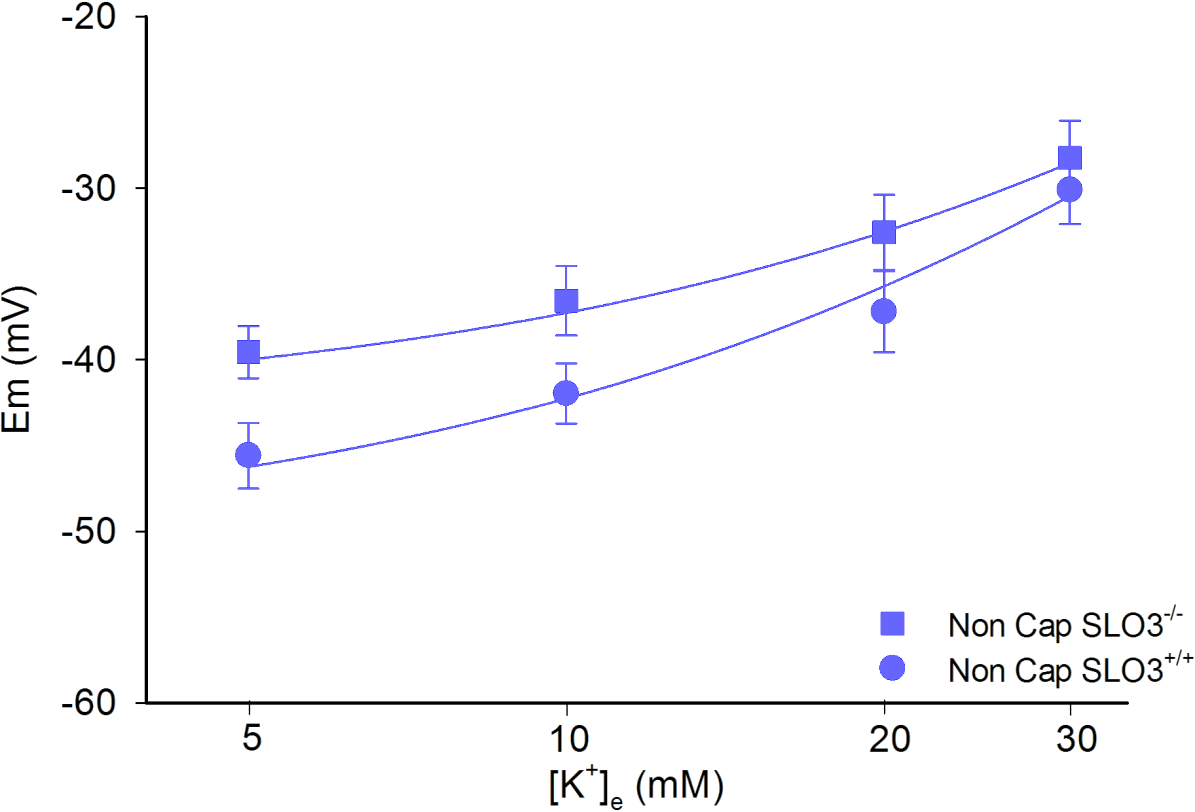

Supplement: Figure S3 — Membrane potential measurements of wild-type and SLO3 mutant sperm in non-capacitated conditions. Curves shown are GHK fits comparing wild-type and SLO3 mutant sperm in non-capacitating conditions. The curves reveal that SLO3 mutant sperm are more depolarized than wild-type in non-capacitating conditions (See text), and this difference is statistically significant (P≤0.05) (to compare the curves we used chi-square test). The curves correspond to mean n = 11 experiments ± S.E.M. See table S1 for membrane potential values. (TIF) [file pone.0060578.s003.tif]

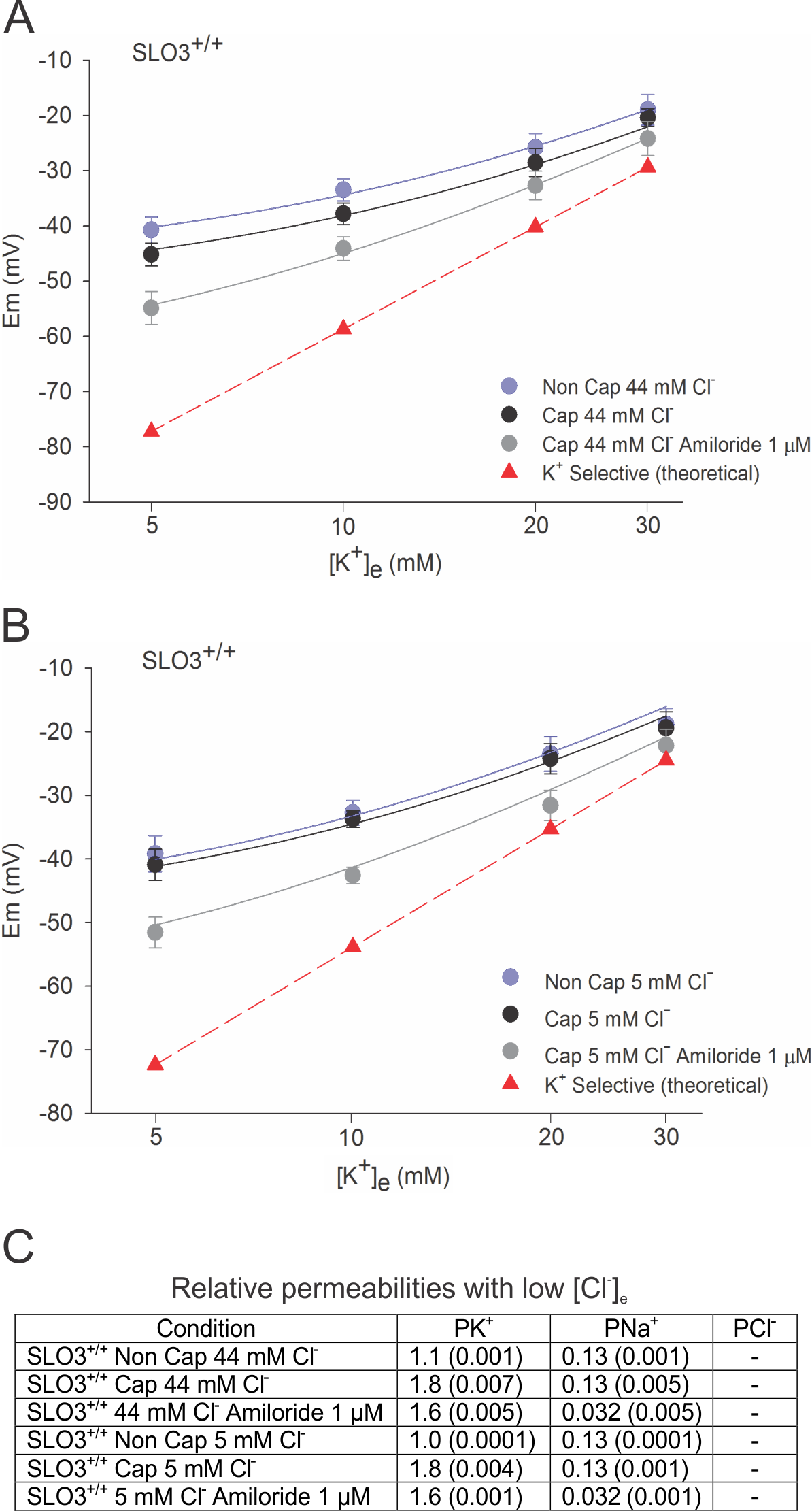

Supplement: Figure S4 — Plots of membrane potential measurements in low [Cl−]e in non-capacitated and capacitated conditions in wild-type sperm. Curves represent GHK fits in wild-type sperm in 44 mM (A) and 5 mM (B) external Cl−. These curves are steeper than those shown in figure 1 where the voltage measurements were made under conditions of normal [Cl−]e. Permeability values predicted by the GHK equation are given in (C). Unlike figure 1, the GHK fits did not require consideration of chloride ion permeability (see text). The curves correspond to mean n = 3 experiments ± S.E.M. See tables S9 and S10 for membrane potential values. (TIF) [file pone.0060578.s004.tif]
