# Supplementary material for: Ion Permeabilities in Mouse Sperm Reveal an External Trigger for SLO3-Dependent Hyperpolarization
Source: PLoS One. 2013 Apr 5;8(4):e60578. doi: 10.1371/journal.pone.0060578 (PMC3618424; doi:10.1371/journal.pone.0060578)
Supplement: Table S1 — Membrane potentials in Non capacitated and Capacitated conditions. Em values obtained at the indicated external K+ concentrations, in wild-type (SLO3+/+) or SLO3 mutant (SLO3− /−) sperm under Non capacitated (Non Cap) and Capacitated (Cap) conditions. Values are given in millivolts (mV) and correspond to mean n = 11 and numbers within brackets correspond to S.E.M. (DOC) [file pone.0060578.s005.doc]

**Table S1. Membrane potentials in Non capacitated and Capacitated conditions.**

| [K^+^]_e_ (mM) | SLO3^+/+^  Non Cap (mV) | SLO3^-/-^  Non Cap (mV) | SLO3^+/+^  Cap (mV) | SLO3^-/-^  Cap (mV) |
| --- | --- | --- | --- | --- |
| 5 | -45.59 (2.57) | -39.55 (1.52) | -63.88 (2.92) | -41.51 (2.01) |
| 10 | -41.96 (1.77) | -36.62 (2.34) | -54.18 (1.99) | -38.83 (1.99) |
| 20 | -37.19 (3.04) | -32.56 (2.18) | -42.61 (2.51) | -34.84 (2.14) |
| 30 | -30.09 (1.98) | -28.22 (2.14) | -33.60 (1.92) | -30.48 (2.28) |
