# Supplementary material for: Ion Permeabilities in Mouse Sperm Reveal an External Trigger for SLO3-Dependent Hyperpolarization
Source: PLoS One. 2013 Apr 5;8(4):e60578. doi: 10.1371/journal.pone.0060578 (PMC3618424; doi:10.1371/journal.pone.0060578)
Supplement: Table S2 — Membrane potentials during different time of capacitation. Em values obtained at the indicated incubation times, in wild-type (SLO3+/+) or SLO3 mutant (SLO3− /−) sperm. Values are given in millivolts (mV) and correspond to mean n = 12 and numbers within brackets correspond to S.E.M. (DOC) [file pone.0060578.s006.doc]

**Table S2. Membrane potentials during different time** of capacitation.

| Time (min) | SLO3^+/+^ (mV) | SLO3^-/-^  (mV) |
| --- | --- | --- |
| 15 | -43.68 (-2.78) | -39.60 (1.38) |
| 30 | -53.03 (-2.49) | -39.65 (2.52) |
| 60 | -63.13 (-1.85) | -39.77 (1.72) |
| 120 | -63.51 (-1.63) | -40.72 (1.08) |
| 180 | -63.18 (-2.83) | -40.13 (2.17) |
