# Supplementary material for: Ion Permeabilities in Mouse Sperm Reveal an External Trigger for SLO3-Dependent Hyperpolarization
Source: PLoS One. 2013 Apr 5;8(4):e60578. doi: 10.1371/journal.pone.0060578 (PMC3618424; doi:10.1371/journal.pone.0060578)
Supplement: Table S3 — Membrane potentials using SLO3 antagonists. Em values obtained at the indicated external K+ concentrations, in wild-type (SLO3+/+) or SLO3 mutant (SLO3− /−) sperm under Capacitated (Cap) conditions in the presence of Ba2+1 mM and Clofilium 50 µM. Values are given in millivolts (mV) and correspond to mean n = 3 and numbers within brackets are S.E.M. (DOC) [file pone.0060578.s007.doc]

**Table S**3. Membrane potentials using SLO3 antagonists.

| [K^+^]_e_ (mM) | SLO3^+/+^ Cap  Ba^2+^ (mV) | SLO3^-/-^ Cap  Ba^2+^ (mV) | SLO3^+/+^ Cap Clofilium (mV) | SLO3^-/-^ Cap Clofilium (mV) |
| --- | --- | --- | --- | --- |
| 5 | -41.08 (2.38) | -36.95 (2.37) | -43.96 (2.38) | -37.44 (2.66) |
| 10 | -38.18 (2.44) | -36.57 (2.55) | -40.55 (2.44) | -37.04 (2.72) |
| 20 | -35.32 (1.99) | -33.29 (1.31) | -37.66 (1.99) | -34.57 (1.59) |
| 30 | -29.84 (2.38) | -29.88 (2.88) | -30.60 (2.38) | -30.47 (2.95) |
