# Supplementary material for: Ion Permeabilities in Mouse Sperm Reveal an External Trigger for SLO3-Dependent Hyperpolarization
Source: PLoS One. 2013 Apr 5;8(4):e60578. doi: 10.1371/journal.pone.0060578 (PMC3618424; doi:10.1371/journal.pone.0060578)
Supplement: Table S4 — Membrane potentials in pH 7. Em values obtained at the indicated external K+ concentrations, in wild-type (SLO3+/+) or SLO3 mutant (SLO3− /−) sperm under Non capacitated (Non Cap) and Capacitated (Cap) conditions in external pH 7. Values are given in millivolts (mV) and correspond to mean n = 4 and numbers within brackets correspond to S.E.M. (DOC) [file pone.0060578.s008.doc]

**Table S**4. Membrane potentials in pH 7

| [K^+^]_e_ (mM) | SLO3^+/+^ Non Cap (mV) | SLO3^-/-^ Non  Cap (mV) | SLO3^+/+^  Cap (mV) | SLO3^-/-^  Cap (mV) |
| --- | --- | --- | --- | --- |
| 5 | -45.13 (2.68) | -38.13 (2.11) | -50.31 (2.24) | -40.13 (1.82) |
| 10 | -39.65 (2.93) | -34.21 (1.98) | -43.65 (2.68) | -36.51 (1.91) |
| 20 | -34.11(2.47) | -28.24 (2.45) | -36.10 (2.74) | -29.19 (1.33) |
| 30 | -29.81 (2.35) | -26.07 (1.54) | -30.13 (2.15) | -26.39 (1.88) |
