# Supplementary material for: Ion Permeabilities in Mouse Sperm Reveal an External Trigger for SLO3-Dependent Hyperpolarization
Source: PLoS One. 2013 Apr 5;8(4):e60578. doi: 10.1371/journal.pone.0060578 (PMC3618424; doi:10.1371/journal.pone.0060578)
Supplement: Table S5 — Membrane potentials in pH 8. Em values obtained at the indicated external K+ concentrations, in wild-type (SLO3+/+) or SLO3 mutant (SLO3− /−) sperm under Non capacitated (Non Cap) and Capacitated (Cap) conditions in external pH 8. Values are given in millivolts (mV) and correspond to mean n = 4 and numbers within brackets correspond to S.E.M. (DOC) [file pone.0060578.s009.doc]

**Table S**5. Membrane potentials in pH 8

| [K^+^]_e_ (mM) | SLO3^+/+^ Non  Cap (mV) | SLO3^-/-^ Non  Cap (mV) | SLO3^+/+^  Cap (mV) | SLO3^-/-^  Cap (mV) |
| --- | --- | --- | --- | --- |
| 5 | -64.47 (2.77) | -43.88 (2.20) | -74.95 (2.76) | -43.81 (2.24) |
| 10 | -52.52 (2.99) | -36.65 (2.34) | -56.89 (2.81) | -36.65 (2.52) |
| 20 | -40.95 (2.17) | -30.39 (2.77) | -43.99 (2.98) | -30.39 (2.76) |
| 30 | -33.19(2.35) | -26.84 (2.80) | -34.26 (2.55) | -26.71 (2.91) |
