# Supplementary material for: Ion Permeabilities in Mouse Sperm Reveal an External Trigger for SLO3-Dependent Hyperpolarization
Source: PLoS One. 2013 Apr 5;8(4):e60578. doi: 10.1371/journal.pone.0060578 (PMC3618424; doi:10.1371/journal.pone.0060578)
Supplement: Table S6 — Membrane potentials with Amiloride and low [Na+]e. Em values obtained at the indicated external K+ concentrations, in wild-type (SLO3+/+) or SLO3 mutant (SLO3− /−) sperm under Non capacitated (Non Cap) and Capacitated (Cap) conditions in the presence of Amiloride 1 µM or in 1 mM external Na+. Values are given in millivolts (mV) and correspond to mean n = 4 and numbers within brackets correspond to S.E.M. (DOC) [file pone.0060578.s010.doc]

**Table S6. Membrane potentials with Amiloride and low [Na^+^]_e_**

| [K^+^]_e_ (mM) | SLO3^+/+^ Non Cap Amiloride (mV) | SLO3^-/-^ Non Cap Amiloride (mV) | SLO3^+/+^ Non Cap  1 mM Na^+^ (mV) | SLO3^-/-^ Non Cap  1 mM Na^+^ (mV) |
| --- | --- | --- | --- | --- |
| 5 | -75.52 (1.82) | -69.03 (2.53) | -76.11 (2.40) | -72.25 (2.99) |
| 10 | -59.48 (2.48) | -51.70 (2.87) | -57.40 (2.65) | -53.79 (2.67) |
| 20 | -42.24 (2.03) | -36.82 (2.08) | -43.24 (2.78) | -40.54 (2.19) |
| 30 | -31.12 (1.86) | -29.74 (1.58) | -31.27 (2.47) | -30.28 (2.09) |
