# Supplementary material for: Ion Permeabilities in Mouse Sperm Reveal an External Trigger for SLO3-Dependent Hyperpolarization
Source: PLoS One. 2013 Apr 5;8(4):e60578. doi: 10.1371/journal.pone.0060578 (PMC3618424; doi:10.1371/journal.pone.0060578)
Supplement: Table S7 — Membrane potentials using mitochondrial un-coupler CCCP. Em values obtained at the indicated external K+ concentrations, in wild-type (SLO3+/+) or SLO3 mutant (SLO3− /−) sperm under Non capacitated (Non Cap) and Capacitated (Cap) conditions in the presence of CCCP 0.5 µM. Values are given in millivolts (mV) and correspond to mean n = 5 and numbers within brackets correspond to S.E.M. (DOC) [file pone.0060578.s011.doc]

**Table S**7. Membrane potentials using mitochondrial un-coupler CCCP

| [K^+^]_e_ (mM) | SLO3^+/+^ Non  Cap (mV) | SLO3^-/-^ Non  Cap (mV) | SLO3^+/+^  Cap (mV) | SLO3^-/-^  Cap (mV) |
| --- | --- | --- | --- | --- |
| 5 | -44.20 (2.22) | -38.86 (1.88) | -63.29 (1.60) | -39.68 (1.57) |
| 10 | -38.99 (2.20) | -34.55 (1.76) | -51.10 (2.31) | -36.87 (1.74) |
| 20 | -33.29 (2.29) | -30.56 (1.90) | -38.22 (2.35) | -31.08 (2.08) |
| 30 | -29.43 (2.47) | -27.22 (2.08) | -33.58 (1.74) | -28.85 (1.94) |
