# Supplementary material for: Ion Permeabilities in Mouse Sperm Reveal an External Trigger for SLO3-Dependent Hyperpolarization
Source: PLoS One. 2013 Apr 5;8(4):e60578. doi: 10.1371/journal.pone.0060578 (PMC3618424; doi:10.1371/journal.pone.0060578)
Supplement: Table S8 — Membrane potentials using mitochondrial un-couplers Antimycin and Oligomycin. Em values obtained at the indicated external K+ concentrations, in wild-type (SLO3+/+) or SLO3 mutant (SLO3− /−) sperm under Non capacitated (Non Cap) and Capacitated (Cap) conditions in the presence of Antimycin 1 µM and Oligomycin 0.5 µM. Values are given in millivolts (mV) and correspond to mean n = 3 and numbers within brackets correspond to S.E.M. (DOC) [file pone.0060578.s012.doc]

**Table S8. Membrane potentials** **using mitochondrial un-couplers Antimycin and Oligomycin.**

| [K^+^]_e_ (mM) | SLO3^+/+^ Non Cap (mV) | SLO3^-/-^ Non Cap (mV) | SLO3^+/+^  Cap (mV) | SLO3^-/-^  Cap (mV) |
| --- | --- | --- | --- | --- |
| 5 | -41.18 (1.74) | -39.81 (2.48) | -62.21 (1.94) | -41.75 (1.95) |
| 10 | -35.94 (1.78) | -35.21 (1.81) | -49.01 (1.61) | -35.01 (1.87) |
| 20 | -29.98 (1.83) | -29.48 (1.44) | -35.55 (2.17) | -30.58 (1.84) |
| 30 | -27.19 (1.54) | -26.91 (1.07) | -30.71 (1.79) | -27.71 (1.97) |
