# Supplementary material for: Ion Permeabilities in Mouse Sperm Reveal an External Trigger for SLO3-Dependent Hyperpolarization
Source: PLoS One. 2013 Apr 5;8(4):e60578. doi: 10.1371/journal.pone.0060578 (PMC3618424; doi:10.1371/journal.pone.0060578)
Supplement: Table S9 — Membrane potentials in SLO3+/+ using 44 mM Cl− external. Em values obtained at the indicated external K+ concentrations, in wild-type (SLO3+/+) sperm under Non capacitated (Non Cap) and Capacitated (Cap) conditions using 44 mM external Cl−. Values are given in millivolts (mV) and correspond to mean n = 3 and numbers within brackets correspond to S.E.M. (DOC) [file pone.0060578.s013.doc]

**Table S9. Membrane potentials in SLO3^+/+^ using 44 mM Cl^-^** external

| [K^+^]_e_ (mM) | Non Cap (mV) | Cap (mV) | Cap Amiloride (mV) |
| --- | --- | --- | --- |
| 5 | -40.74 (2.37) | -45.17 (2.07) | -54.87 (2.99) |
| 10 | -33.47 (1.98) | -37.80 (1.92) | -44.09 (2.14) |
| 20 | -25.84 (2.54) | -28.52 (2.55) | -32.69 (2.58) |
| 30 | -18.97 (2.81) | -20.39 (1.63) | -24.18 (3.07) |
