# Supplementary material for: Ion Permeabilities in Mouse Sperm Reveal an External Trigger for SLO3-Dependent Hyperpolarization
Source: PLoS One. 2013 Apr 5;8(4):e60578. doi: 10.1371/journal.pone.0060578 (PMC3618424; doi:10.1371/journal.pone.0060578)
Supplement: Table S10 — Membrane potentials in SLO3+/+ using 5 mM Cl− external. Em values obtained at the indicated external K+ concentrations, in wild-type (SLO3+/+) sperm under Non capacitated (Non Cap) and Capacitated (Cap) conditions using 5 mM external Cl−. Values are given in millivolts (mV) and correspond to mean n = 3 and numbers within brackets correspond to S.E.M. (DOC) [file pone.0060578.s014.doc]

**Table S10. Membrane potentials in SLO3^+/+^ using 5 mM Cl^-^ external**

| [K^+^]_e_ (mM) | Non Cap (mV) | Cap (mV) | Cap Amiloride (mV) |
| --- | --- | --- | --- |
| 5 | -39.18 (2.85) | -40.89 (2.43) | -50.05 (2.15) |
| 10 | -32.73 (1.94) | -33.72 (1.30) | -41.62 (1.85) |
| 20 | -23.52 (2.71) | -24.24 (2.37) | -30.08 (2.63) |
| 30 | -18.85 (2.55) | -19.43 (2.55) | -21.15 (2.51) |
